# Multilevel genomic, transcriptomic, and epidemiologic evidence linking diabetic retinopathy to Alzheimer disease

**DOI:** 10.7189/jogh.16.04311

**Published:** 2026-09-04

**Authors:** Jing Li, Qian Liu, Qian Ma, Ting Shen, Jiao Xia, Xiongyi Yang, Kun Liu, Na Li, Biao Yan

**Affiliations:** 1 Eye Institute and Department of Ophthalmology, Eye & ENT Hospital of Fudan University, Shanghai, China; 2 Department of Ophthalmology, Shanghai General Hospital, Shanghai Jiao Tong University School of Medicine, Shanghai, China; 3 Department of Ophthalmology, General Hospital of Ningxia Medical University, Yinchuan, China

**Keywords:** diabetic retinopathy, Alzheimer disease, Mendelian randomisation, genetic correlation, single-cell transcriptomics

## Abstract

**Background:**

Diabetic retinopathy (DR) and Alzheimer disease (AD) share metabolic and vascular dysfunctions, but the extent to which they reflect overlapping genetic susceptibility and neurovascular-metabolic regulatory pathways remains unclear. We combined multi-omics analyses with population-based data to examine the genetic convergence, cellular pathways, and longitudinal association between DR and AD.

**Methods:**

We performed a two-sample Mendelian randomisation (MR) to estimate the association between genetically predicted DR liability and AD risk. We used Bayesian colocalisation analysis to identify shared genomic loci, and summary-data-based MR (SMR) to detect expression-mediated genes jointly associated with DR and AD. We analysed single-cell RNA sequencing data to characterise shared cellular features and related biological pathways. We also conducted an MR-based mediation analysis to explore whether lipid-related, metabolic, or inflammatory traits mediated the observed DR-AD association, and a longitudinal analysis of the UK Biobank cohort to assess the association between DR and incident AD.

**Results:**

With the MR analysis, we found that genetically predicted liability to DR was associated with a modest increase in AD risk. Colocalisation analysis supported a shared genetic signal. We identified three genes with shared expression-mediated associations across DR and AD through SMR. Functional enrichment analyses revealed partially overlapping neurovascular and metabolic pathways. Using MR-based mediation analysis, we found no significant intermediary traits linking DR and AD. Findings from the UK Biobank cohort were directionally consistent with the genetic analyses.

**Conclusions:**

Genetic liability to DR is associated with an increased risk of AD and is accompanied by shared expression-mediated effects and convergent neurovascular-metabolic pathways. These findings support the possibility that DR may serve as a clinically accessible indicator of increased neurodegenerative vulnerability.

Alzheimer disease (AD) is among the most significant global health challenges of the 21st century, affecting an estimated 55.2 million individuals worldwide, with the number of affected people projected to increase to nearly 78 million by 2030 [1]. Main AD characteristics include progressive cognitive decline and hallmark neuropathological features, including amyloid-β plaques and neurofibrillary tangles [2,3]. Beyond its classical neuropathological hallmarks, the pathogenesis of AD is increasingly recognised to involve chronic neuroinflammation, mitochondrial dysfunction, metabolic imbalance, and cerebrovascular dysfunction [4,5]. Clinically, patients exhibit a range of symptoms ranging from early mood disturbances (*e.g.* depression and anxiety) to advanced cognitive impairment and neuropsychiatric manifestations [6]. Although considerable progress has been made in understanding the disease, effective therapies capable of slowing or reversing its progression are still lacking, and diagnosis is often made only after substantial neuronal damage has occurred [7]. Notably, pathological changes are believed to develop long before clinical symptoms become apparent, emphasising the importance of identifying accessible biomarkers and better defining modifiable risk factors linked to disease development [8,9].

The retina has increasingly been viewed as a potential non-invasive marker for neurodegenerative diseases, including AD. Because the retina shares developmental and neurovascular features with the brain, AD-related pathological changes, including amyloid-β deposition, neuronal loss, and vascular abnormalities, have also been detected in retinal tissue [10]. These findings have driven growing interest in retinal imaging as a possible approach for identifying early neurodegenerative changes [11–13]. Meanwhile, diabetic retinopathy (DR), a major microvascular complication of diabetes and a leading cause of vision loss worldwide, is characterised by progressive retinal vascular damage [14]. Notably, DR and AD share several features, including metabolic imbalance, vascular dysfunction, inflammation, oxidative stress, and altered lipid metabolism. However, whether these similarities reflect shared biological mechanisms or broader systemic metabolic dysfunction remains unclear. Evidence regarding the relationship between DR and AD also remains inconsistent, with some studies supporting a direct link, while others suggest that both conditions may be influenced by common systemic factors such as glucose dysregulation and vascular impairment [15].

To address these questions, we used Mendelian randomisation (MR), which applies genetic variants as instrumental variables to reduce confounding and reverse causation [16]. Using genome-wide association study (GWAS) data, we examined the association between genetically predicted liability to DR and AD risk. We further combined colocalisation analysis, summary-data-based MR (SMR), single-cell RNA sequencing (scRNA-seq), functional enrichment analysis, MR-based mediation analysis, and longitudinal cohort analysis to explore shared genetic signals, cellular features, and biological pathways linking the two diseases.

## METHODS

### Study population and disease definition

We used UK Biobank data, including participants’ baseline demographic, lifestyle, physical, and biomarker data from standardised questionnaires, clinical assessments, and linked medical records. Responses of ‘Do not know’ or ‘Prefer not to answer’ were treated as missing. We excluded participants with prevalent dementia at baseline, inconsistent clinical records (*e.g.* DR without diabetes), or missing covariates, leaving 308,125 individuals for analysis (Figure S1 in the **Online Supplementary Document**). Incident AD was identified using data from linked hospital, primary care, death registry, and self-reported medical conditions, with AD defined according to ICD-10 codes F00 and G30. Follow-up extended from baseline until incident AD, death, loss to follow-up, or 31 December 2024. The DR exposure was defined using ICD-10 codes H36.0 and E10.3–E14.3. We selected age, sex, ethnicity, body mass index (BMI), systolic and diastolic blood pressure, smoking status, alcohol consumption, glycated haemoglobin (HbA1c), high-density lipoprotein (HDL), and low-density lipoprotein (LDL) as covariates before analysis.

### MR analysis

We investigated the potential causal effect between genetically predicted liability to DR and AD using *R* packages ‘TwoSampleMR’, ‘MendelianRandomization’, and ‘gwasglue’ [17]. We selected instrumental variables from genome-wide significant single-nucleotide polymorphisms (SNPs) (*P* < 5 × 10^−8^) and clumped them using a linkage disequilibrium (LD) threshold of *r*^2 ^< 0.001 within a 10,000 kb window based on 1000 Genomes European reference data. Instrument strength was assessed by calculating *R*^2^ and *F*-statistics, and SNPs with *F *< 10 were excluded. We harmonised exposure and outcome data sets using the ‘TwoSampleMR’ pipeline to align effect alleles between data sets. Ambiguous palindromic SNPs with intermediate allele frequencies were excluded during harmonisation. We obtained primary MR estimates using the inverse-variance weighted method [18], with multiple-testing correction using the false discovery rate (FDR) method. For sensitivity analyses, we used MR-PRESSO for outlier detection [19], Cochran’s Q test for heterogeneity (with *P* > 0.05 indicating no evidence of substantial heterogeneity) [20], MR-Egger regression for directional pleiotropy (with *P* > 0.05 indicating no evidence of directional pleiotropy) [21], and leave-one-out validation [22]. To further evaluate whether the observed association was driven by *APOE*-related variants, we excluded SNPs in LD (*r*^2 ^> 0.1, based on the 1000 Genomes European reference panel) with *APOE* variants using the ‘LDlinkR’ package in *R*, as well as SNPs within the extended *APOE* genomic region (chr19: 44.5-46 Mb, GRCh37), given the strong pleiotropic effects of *APOE* on AD risk.

### Colocalisation and pairwise LD analysis

Colocalisation analysis investigates whether two traits share the same causal variant within a specific genomic locus, providing evidence for potentially shared regulatory architecture within specific genomic regions. We identified shared causal variants between DR and AD using the ‘coloc’ *R* package to integrate GWAS summary statistics. Analyses focused on genome-wide significant loci (*P* < 5 × 10^−8^), with posterior probability for hypothesis 4 (PP.H4) > 0.8 defining robust colocalisation [23]. We used the default prior probabilities implemented in the ‘coloc’ package unless otherwise specified.

### SMR analysis

The SMR analysis combines GWAS summary statistics with expression quantitative trait loci (eQTL) data to identify genes whose expression levels are potentially causally associated with complex traits or diseases. To investigate whether gene expression mediates the shared genetic architecture between DR and AD, we performed SMR analysis using the SMR software [24]. We obtained brain eQTL data from the PsychENCODE Consortium (cis-eQTL, corrected for 50 PEER factors), primarily derived from human cortical tissues. Peripheral blood eQTL data were obtained from the Genotype-Tissue Expression project (GTEx v8 whole blood). We used the heterogeneity in dependent instruments (HEIDI) test to distinguish pleiotropy from linkage and considered genes with SMR *P-*values passing multiple-testing correction and with test *P* > 0.05. We applied FDR correction to SMR *P*-values.

### scRNA-seq and functional enrichment analysis

We processed scRNA-seq data sets using the ‘Seurat’ *R* package. To identify convergent molecular signatures while reducing cross-data set technical variability, we performed independent cell-type-specific differential expression analyses within each data set using the Seurat FindMarkers function. We then identified shared differentially expressed genes (DEGs) as genes showing significant dysregulation in the same direction across broadly analogous functional cell populations in both DR and AD data sets. Shared DEGs were visualised using Venn diagrams and heatmaps across analogous neurovascular and neuroglial cell populations in DR and AD. Functional enrichment analysis provides insight into the biological processes and pathways associated with a given set of genes [25].

### MR–based mediation analysis

To investigate potential mediators linking genetically predicted liability to DR and AD, we performed a two-step MR-based mediation analysis. We evaluated a range of lipid-related, metabolic, and inflammatory traits, including HDL, LDL, triglycerides, polyunsaturated fatty acids, omega-6 and omega-3 fatty acids, monounsaturated fatty acids, saturated fatty acids, BMI, adiponectin, apolipoprotein B, fasting blood glucose, HbA1c, proinsulin, and glutamate.

The mediation process consisted of three sequential steps: estimating the causal effect of DR on each potential mediator; assessing the causal effect of each mediator on AD; and evaluating the mediation proportion and statistical significance. We estimated the indirect effect using the product-of-coefficients method and calculated the mediation proportion as the ratio of the indirect effect to the total effect. We estimated standard errors using the delta method.

### Statistics

We summarised the baseline characteristics by DR status. We presented continuous variables as mean (standard deviation), and categorical variables as counts (%). We assessed group differences using standardised mean differences (SMDs). We evaluated associations between DR and incident AD using Cox proportional hazards models. In the primary analysis, DR was treated as a time-varying exposure to account for incident DR during follow-up. A secondary analysis considered DR as a baseline exposure, defined by the presence of DR at or before study entry. Models were adjusted for age, sex, ethnicity, BMI, systolic and diastolic blood pressure, smoking status, alcohol consumption, HbA1c, LDL, and HDL.

To reduce baseline differences between participants with and without DR, we used inverse probability of treatment weighting (IPTW) based on stabilised propensity scores. We estimated propensity scores using demographic, metabolic, and lifestyle-related variables, including diabetes duration. Because DR occurs in the setting of diabetes, IPTW analyses were limited to participants with diabetes at baseline to improve comparability between groups. We estimated hazard ratios (HRs) and 95% confidence intervals (CIs) using weighted Cox models. We handled missing covariates using multiple imputation by chained equations (m = 20), combining estimates using Rubin’s rules. E-values were calculated to evaluate the potential influence of unmeasured confounding.

Sensitivity analyses included additional adjustment for urinary albumin-to-creatinine ratio, exclusion of AD cases occurring within the first 1–2 years of follow-up, landmark analyses at different time points (0.5, 1, 2, 3, and 5 years), alternative outcome definitions using all-cause dementia, single imputation for missing covariates, and further adjustment for diabetes status and duration. We evaluated the proportional hazards assumption using Schoenfeld residuals. All analyses were performed in *R*, version 4.3.2 (R Core Team, Vienna, Austria).

## RESULTS

### Data resource

For the primary MR analysis, we treated DR as the exposure and AD as the outcome. The DR GWAS data sets included 29,668 cases and 402,541 controls, while the multi-population DR data set comprised 47,590 cases and 563,698 controls. The AD data sets included 24,087 clinically diagnosed late-onset AD cases, 47,793 individuals with a family history of AD, and 383,378 European-ancestry controls (Table S1 in the **Online Supplementary Document**). For the mediation analyses, we used metabolic, lipid, and inflammatory traits from the IEU OpenGWAS database, including HDL, LDL, triglycerides, fatty acids, BMI, adiponectin, apolipoprotein B, and glycaemic traits.

Participants with DR tended to be older and had higher BMI, SBP, and HbA1c levels than those without DR (**Table 1**). They were also more likely to be of Asian or Black ethnicity, and alcohol consumption patterns differed slightly between groups. These differences reflect a higher cardiometabolic burden in individuals with DR at baseline. SMDs further highlighted key between-group differences, supporting the need for multivariable adjustment in the analyses.

**Table 1:** Baseline clinical characteristics of participants by DR status*

	DR (n = 2,874)	No DR (n = 305,251)	SMD
Age in years*	60.3 (6.7)	56.0 (8.2)	0.6
**Sex†**			0.4
Male	1,853 (64.5)	138,180 (45.3)	
Female	1,021 (35.5)	167,071 (54.7)	
**Race†**			0.3
White	2,472 (86.0)	291,336 (95.4)	
Asian	226 (7.9)	5,898 (1.9)	
Black	95 (3.3)	3,881 (1.3)	
Other	81 (2.8)	4,136 (1.4)	
BMI*	31.4 (5.8)	27.0 (4.4)	0.9
SBP*	145.8 (19.1)	139.1 (19.6)	0.3
DBP*	79.8 (11.0)	82.1 (10.7)	0.2
**Smoking status†**			0.1
No	1,016 (35.4)	124,330 (40.7)	
Yes	1,858 (64.6)	180,921 (59.3)	
**Drinking status†**			0.4
Never	298 (10.4)	12,009 (3.9)	
Past	247 (8.6)	9,673 (3.2)	
Current	2,329 (81.0)	283,569 (92.9)	
HbA1c*	59.7 (16.0)	34.8 (4.0)	2.1
HDL*	1.2 (0.4)	1.5 (0.4)	0.7
LDL*	2.6 (0.7)	3.6 (0.8)	1.2

BMI – body mass index, DBP – diastolic blood pressure, DR – diabetic retinopathy, HbA1c – glycated haemoglobin, HDL – high-density lipoprotein, LDL – low-density lipoprotein, SBP – systolic blood pressure, SMD – standardised mean differences

*Values are presented as mean (standard deviation).

†Values are presented as n (%).

### Causal inference of DR on AD *via* MR analysis and colocalisation analysis

To examine the potential causal relationship between genetically predicted liability to DR and AD, we conducted a two-sample MR analysis, treating DR as the exposure and AD as the outcome (Table S2 in the **Online Supplementary Document**). Inverse-variance weighted analysis indicated a positive association between genetically predicted liability to DR and AD risk (OR = 1.012; 95% CI = 1.004–1.020, *P* = 0.002) (Figure S2 in the **Online Supplementary Document**). Sensitivity analyses supported the robustness of this finding, showing no significant heterogeneity (Cochran’s Q *P* > 0.05), no evidence of directional pleiotropy (MR-Egger intercept *P* > 0.05), and no influential outliers (MR-PRESSO global test *P* > 0.05) (**Table 2**).

**Table 2:** Sensitivity validation of MR assumptions

		Cochran’s Q test		MR-Egger intercept test		MR-PRESSO
**Exposure**	**Outcome**	**Q statistic**	***P*-value**	**Intercept**	***P*-value**	***P*-value**
DR	AD	119.724	0.124	−0.002	0.064	0.089
DR (multi-population)	AD	100.659	0.021	−0.001	0.475	0.023

AD – Alzheimer disease, DR – diabetic retinopathy, MR – Mendelian randomisation

We also performed a reverse MR analysis, considering AD as the exposure and DR as the outcome. This analysis found no evidence for a causal effect of AD on DR (OR = 0.876; 95% CI = 0.669–1.147, *P* = 0.336), suggesting that the association is likely unidirectional.

To further investigate, we expanded the analysis to a multi-population DR GWAS data set, including individuals of African American or Afro-Caribbean, East Asian, European, and Hispanic or Latin American ancestry. The inverse-variance weighted analysis remained significant, indicating a positive association between genetically predicted DR liability and AD risk (OR = 1.013; 95% CI = 1.003–1.023, *P* = 0.008). Notably, the multi-population analysis showed significant heterogeneity (**Table 2**), which may reflect ancestry-specific genetic architecture, allele-frequency heterogeneity, or population-specific pleiotropy.

Colocalisation analysis identified a genome-wide significant shared locus on chromosome 19 (PP.H4 = 0.938), with rs429358, located near *APOE*, showing the strongest evidence (PP.H4 = 0.998) (Figure S3 in the **Online Supplementary Document**). The co-localised gene *APOE* is well known for its biological relevance to AD [26]. To reduce potential bias, we excluded variants within the *APOE* region as well as other loci identified as potentially pleiotropic through LDtrait annotation (Table S3 in the **Online Supplementary Document**). After these exclusions, the association between genetically predicted DR liability and AD risk remained directionally consistent and statistically significant (OR = 1.015; 95% CI = 1.006–1.024, *P* = 0.001). Sensitivity analyses also supported these findings (Table S4 in the **Online Supplementary Document**), suggesting the observed association was not driven solely by variants near *APOE*.

### Identification of shared genetically mediated genes between DR and AD

Using SMR analysis that integrates DR and AD GWAS data with cortical eQTL data from the PsychENCODE Consortium, we identified two genes that remained significantly associated with both diseases after FDR correction: *SNX32*, significantly associated to both DR (FDR-adjusted *P* = 1.52 × 10^−4^) and AD (FDR-adjusted *P* = 6.90 × 10^−3^) and *FIBP*, also significant for both DR (FDR-adjusted *P* = 1.11 × 10^−3^) and AD (FDR-adjusted *P* = 1.69 × 10^−2^) (Figure S4, Panels A and B in the **Online Supplementary Document**). Using whole blood eQTL data from the Genotype-Tissue Expression project, we found *CTSH* also to be significantly associated with both DR (FDR-adjusted *P* = 1.64 × 10^−2^) and AD (FDR-adjusted *P* = 3.09 × 10^−2^) (Figure S4, Panels C and D in the **Online Supplementary Document**). All identified genes passed the heterogeneity in dependent instruments test (*P* > 0.05), supporting a pleiotropic or potentially causal association rather than LD between distinct variants. Notably, *SNX32* and *FIBP* showed opposite effect directions between DR and AD. This suggests that higher expression of these genes may play different roles in retinal *vs* neurodegenerative contexts. In contrast, *CTSH* exhibited concordant effects in both DR and AD, with increased expression associated with higher disease risk in both conditions. Detailed information is provided (Table S5 in the **Online Supplementary Document**).

### Functional analysis of shared DEGs between DR and AD

**Figure 1:**
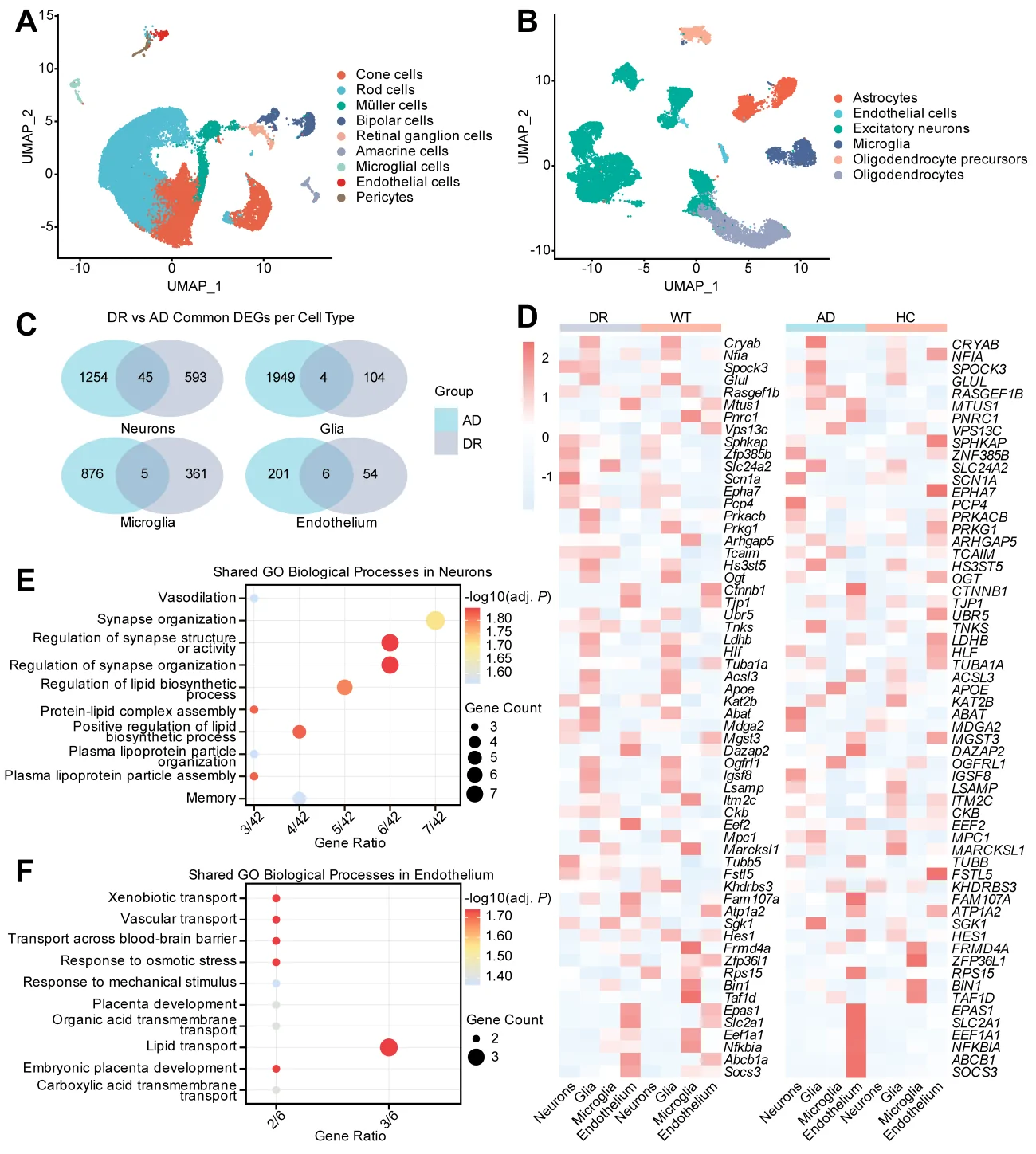
Shared DEGs identified from scRNA-seq data of DR and AD. **Panel A.** UMAP projection of DR single-cell transcriptomic data: major retinal cell populations. **Panel B.** UMAP projection of AD single-cell transcriptomic data: major brain cell populations. **Panel C.** Shared DEGs across corresponding cell types in DR and AD. **Panel D.** Heatmap displaying expression patterns of shared DEGs between DR and AD across relevant cell types. **Panel E.** GO enrichment analysis of shared DEGs in neurons between DR and AD. **Panel F.** GO enrichment analysis of shared DEGs in endothelial cells between DR and AD. AD – Alzheimer disease, DEG – differentially expressed gene, DR – diabetic retinopathy, GO – gene ontology, HC – healthy control, scRNA-seq – single-cell RNA sequencing, UMAP – Uniform Manifold Approximation and Projection, WT – wild type.

To investigate potential biological mechanisms underlying the genetic overlap between DR and AD, we performed differential expression analyses using scRNA-seq data sets from both diseases (**Figure 1**, Panels A and B). For each cell type, we compared disease and control groups to identify DEGs.

We then established cross-disease correspondences between cell types to enable comparison. We mapped excitatory neurons in AD to major retinal neuronal subtypes in DR, including cones, rods, bipolar, amacrine, and ganglion cells. Glial cells in AD (astrocytes, oligodendrocytes, and oligodendrocyte precursor cells) corresponded to Müller cells in DR; microglia were directly matched, and endothelial and pericyte cells in DR were paired with endothelial cells in AD. Several DEGs were shared between these corresponding cell types (**Figure 1**, Panel C). The heatmap indicates that these common DEGs exhibit similar expression patterns across DR and AD (**Figure 1**, Panel D).

Next, GO enrichment analysis revealed that these shared DEGs were enriched in pathways related to synapse organisation, vascular regulation (including vasodilation and transport across the blood–brain barrier), and lipid metabolism, such as lipoprotein particle assembly and lipid biosynthesis (**Figure 1**, Panels E and F).

### MR–based mediation analysis of DR and AD

**Figure 2:**
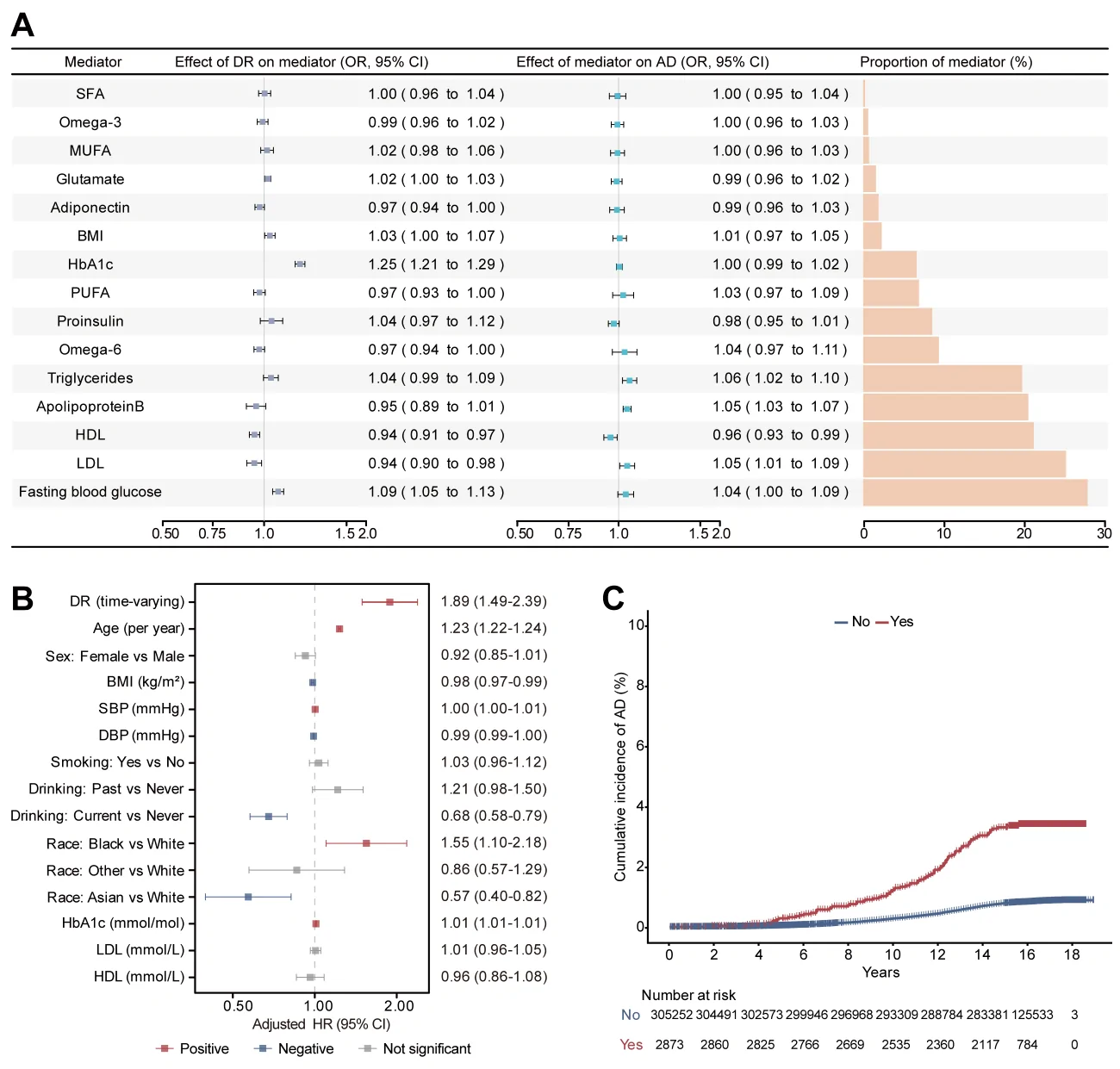
MR-based mediation and survival analyses. **Panel A.** Results of MR-based mediation analyses. **Panel B.** Forest plot of adjusted time-varying HRs for incident AD associated with DR. **Panel C.** Cumulative incidence curve of AD over follow-up time (years). The HRs are plotted on a log scale. AD – Alzheimer disease, BMI – body mass index, DBP – diastolic blood pressure, DR – diabetic retinopathy, HbA1c – glycated haemoglobin, HDL – high-density lipoprotein, HR – hazard ratio, LDL – low-density lipoprotein, MUFA – monounsaturated fatty acids, PUFA – polyunsaturated fatty acids, SBP – systolic blood pressure, SFA – saturated fatty acids.

To explore potential mediators linking genetically predicted liability to DR and AD, we conducted a two-step MR analysis, considering metabolic and inflammatory traits as potential intermediates (**Figure 2**, Panel A; Tables S6 and S7 in the **Online Supplementary Document**).

We observed causal associations between DR and several metabolic traits, including fasting blood glucose, LDL, HDL, BMI, HbA1c, glutamate, adiponectin, and omega-6 fatty acids. When AD was treated as the outcome, apolipoprotein B, LDL, HDL, and triglycerides showed causal effects on AD risk. None of the mediation effects reached statistical significance, although the effects of HDL (*P* = 0.051) and LDL (*P* = 0.075) were close to the threshold for significance.

### Association between DR and incident AD in the UK Biobank cohort

Over a median follow-up of 15.3 years, 2,829 participants developed AD. In the primary analyses using time-varying exposure, DR was associated with an increased risk of AD (HR = 1.89; 95% CI = 1.49–2.39, *P* = 1.06 × 10^−7^) (**Figure 2**, Panel B). The E-value was 2.47, implying that only relatively strong unmeasured confounding could negate the observed association. A similar association was observed when DR was modelled as a baseline (fixed) exposure (HR = 1.88; 95% CI = 1.50–2.37, *P* = 6.66 × 10^−8^) (Table S8 in the **Online Supplementary Document**). Cumulative incidence curves also showed a higher risk of AD among participants with baseline DR compared with those without DR (**Figure 2**, Panel C).

To account for potential confounding by baseline metabolic differences, we performed IPTW restricted to participants with diabetes at baseline (n = 23,593). After balancing age, sex, ethnicity, BMI, blood pressure, smoking, alcohol consumption, HbA1c, lipids, and diabetes duration (all SMDs <0.1) (Figure S5 and Table S9 in the **Online Supplementary Document**), 21,566 effective samples remained, and DR stayed significantly associated with incident AD (HR = 1.38; 95% CI = 1.09–1.75, *P* = 0.008). The corresponding E-value was 2.10, suggesting that a substantial unmeasured confounder would be required to explain away the association.

Sensitivity analyses consistently supported the primary findings. Adjustment for urinary albumin-to-creatinine ratio in the subset with available data did not materially change the results. In landmark analyses at 0.5–5 years, participants who developed DR before each landmark had a higher subsequent risk of AD, with adjusted HRs ranging from 2.02 to 2.18. A 2-year landmark analysis confirmed higher cumulative incidence of AD among participants with DR (Figure S6 in the **Online Supplementary Document**).

Additional sensitivity analyses, including lag exclusion, alternative dementia definitions, and single imputation, yielded effect estimates that were generally consistent with those of the primary model (Tables S10–12 in the **Online Supplementary Document**). In analyses restricted to participants with diabetes and further adjusted for diabetes status and duration, the association was attenuated and no longer statistically significant, although the effect direction remained consistent. In addition, Schoenfeld residual diagnostics did not indicate major violations of the proportional hazards assumption, supporting the robustness of the Cox regression models.

## DISCUSSION

We performed an integrative analysis combining genetic, transcriptomic, single-cell, and longitudinal cohort data to investigate the relationship between DR and AD. Our findings provide evidence for a modest but reproducible association between genetically predicted liability to DR and susceptibility to AD. Using MR analyses, we found a positive association between genetically predicted liability to DR and AD risk, while sensitivity analyses showed relatively stable estimates without clear evidence of substantial pleiotropy in the primary European ancestry analysis.

To further explore the potential genetic basis underlying this relationship, we conducted colocalisation analysis and identified a shared locus near the *APOE* region on chromosome 19, centred on rs429358. Because *APOE* is strongly associated with AD and exhibits broad pleiotropic effects, variants within the *APOE* region and potential pleiotropic variants identified through LDtrait annotation were excluded from the instrumental variables, and MR analysis was repeated. The association remained significant, suggesting the observed relationship was not driven exclusively by local *APOE*-proximal genetic effects. We thus next examined whether shared gene expression signals may contribute to the genetic overlap between DR and AD.

To test whether gene expression underlies this relationship, we integrated GWAS and eQTL data sets using SMR analysis and identified three genes with shared genetically regulated expression signals in DR and AD: the brain-enriched genes *SNX32* and *FIBP*, and the blood-derived gene *CTSH*. The gene *SNX32* belongs to the sorting nexin family and is involved in endosomal trafficking and membrane protein recycling, processes closely linked to neuronal homeostasis and neurodegeneration [27]. The gene *FIBP* encodes a fibroblast growth factor-binding protein associated with FGF1 signalling [28], suggesting a possible role for growth factor-related pathways in the overlap between DR and AD. Lastly, *CTSH* encodes a lysosomal protease implicated in immune regulation and inflammatory responses [29]. Interestingly, *SNX32* and *FIBP* showed opposite effects between DR and AD, whereas *CTSH* showed concordant positive associations in both conditions. These findings suggest that the relationship between DR and AD may involve multiple heterogeneous molecular processes rather than a single common pathway. Therefore, these genes should be considered preliminary candidate signals requiring further experimental validation rather than established mechanistic mediators of DR-AD crosstalk.

To further define the cellular context underlying this relationship, we analysed scRNA-seq data sets from DR and AD. Differential expression analyses identified overlapping DEGs across retinal and brain cell populations, suggesting partially convergent molecular responses between retinal microvascular injury and neurodegeneration. Functional enrichment analysis highlighted pathways related to lipid metabolism, neurovascular regulation, and transmembrane transport pathways. Disturbances in lipid metabolism contribute to retinal vascular injury and inflammation in DR [30], while impaired cholesterol homeostasis has also been linked to amyloid-beta accumulation, neuroinflammation, and neuronal dysfunction in AD [31]. These observations are consistent with previous studies implicating lipid dysregulation in both retinal microvascular disease and neurodegenerative disorders [32–35], supporting the possibility that DR and AD may share broader neurovascular and metabolic vulnerability pathways.

We also examined whether metabolic or inflammatory traits mediate the association between DR and AD using two-step MR-based mediation analyses. Although several genetically predicted traits, including apolipoprotein B, HDL, and LDL, showed associations with either DR or AD individually, none significantly mediated the relationship between the two diseases. This may partly reflect limited statistical power and the methodological limitations inherent to MR-based mediation approaches, particularly when effect sizes are modest. These findings also suggest that the shared relationship between DR and AD may involve more complex or distributed biological mechanisms not fully captured by individual circulating traits.

In the UK Biobank cohort, longitudinal analyses further supported an association between DR and incident AD after adjustment for multiple vascular and metabolic risk factors, which was consistent with the MR findings. Restricted to participants with diabetes, IPTW analyses showed that the association between DR and AD remained after balancing measured diabetes-related confounders, although the estimated effect size was attenuated compared with the primary analysis (HR = 1.38). Additional analyses within the diabetic subgroup, with further adjustment for diabetes duration and related metabolic factors, showed a similar direction of association, although statistical significance was no longer retained. This likely reflects reduced statistical power and the close correlation between DR and diabetes severity. Overall, these findings suggest that DR may serve as a clinically accessible marker of increased AD risk and may reflect shared microvascular and metabolic disturbances linking retinal and neurodegenerative disease.

Nevertheless, several limitations should be acknowledged. First, although the MR analyses suggested an association between genetically predicted DR liability and AD risk, they cannot establish a direct causal relationship. In addition, the genetic instruments for DR were mainly derived from adult populations and likely reflect type 2 diabetes-related retinopathy. As a result, these variants may represent a broader susceptibility to diabetes-related retinal microvascular injury rather than a strictly subtype-specific DR phenotype. Although we conducted primary MR analyses using ancestry-matched European data sets, heterogeneity across multi-population analyses suggests the association may differ by ancestry and may be influenced by population-specific genetic architecture, LD differences, and allele-frequency heterogeneity. Second, because DR develops as a microvascular complication of diabetes, the selected genetic instruments may partly reflect diabetes severity and cumulative glycaemic exposure rather than DR-specific pathological mechanisms alone. Although we performed *APOE*-region exclusion and IPTW analyses to reduce potential confounding, we cannot completely rule out residual confounding from unmeasured metabolic or vascular factors.

In addition, disease ascertainment may have introduced both misclassification and detection bias. The ICD-coded DR in the UK Biobank likely captures predominantly clinically recognised or more severe disease and may underrepresent milder forms of retinopathy identified through screening programmes. Likewise, AD ascertainment based on hospital records, primary care data, death registries, and self-reported information may fail to identify mild or undiagnosed cases. Furthermore, individuals with diabetes, particularly those with clinically recognised DR, may undergo more frequent ophthalmic and medical evaluations, which could increase opportunities for dementia detection and contribute to surveillance-related ascertainment bias.

## CONCLUSIONS

We found that genetically predicted liability to DR is associated with an increased risk of AD, potentially through disruptions in neurovascular and metabolic homeostasis. Tissue-specific expression patterns and enrichment of pathways involved in neurovascular regulation, metabolism, and cellular trafficking further support a complex shared biological basis. Findings from the longitudinal cohort analysis were consistent with the genetic analyses and showed an association between DR and incident AD. Together, these results provide additional evidence for shared neurovascular mechanisms between DR and AD and offer a basis for further mechanistic and translational studies. Future studies integrating longitudinal multi-omics data with experimental validation may help clarify the underlying pathways and inform precision therapeutic methods.

## Additional material


Online Supplementary Document

## Data Availability

**Data availability:** All genome-wide association study (GWAS) summary statistics used in this study are publicly available to qualified researchers. We obtained the data for diabetic retinopathy (DR) (GWAS IDs: GCST90475689 and GCST90479891) and Alzheimer disease (AD) (GWAS ID: GCST007320) from the GWAS Catalog. Single-cell RNA sequencing data set for AD is available from ssREAD. The corresponding data set for DR and additional data sets generated or analysed during the current study are available from the corresponding author upon reasonable request. UK Biobank data are available to approved researchers through the UK Biobank. Data used in this study were accessed under application number 107451.
