# Supplementary material for: Multilevel genomic, transcriptomic, and epidemiologic evidence linking diabetic retinopathy to Alzheimer disease
Source: J Glob Health. 2026 Sep 4;16:04311. doi: 10.7189/jogh.16.04311 (PMC13542801; doi:10.7189/jogh.16.04311)
Supplement: Supplementary file 1 [file jogh-16-04311-s001.pdf]

Supplement to: Li J, Liu Q, Ma Q, Shen T, Xia J, Yang X, Liu K, Li N, Yan B. Multilevel genomic, transcriptomic, and epidemiologic evidence linking diabetic retinopathy to Alzheimer disease. J Glob Health. 2026;16:04311.

Supplementary figures:

Figure S1. Flowchart of study cohort selection.

Figure S2. Causal effect of DR on AD as estimated by MR analysis.

Figure S3. Locus-level comparison of association signals between DR and AD.

Figure S4. SMR locus plots for representative shared genes between DR and AD.

Figure S5. Love plot showing covariate balance before and after inverse probability of treatment weighting in the diabetes-restricted cohort.

Figure S6. Cumulative incidence curves of AD from the 2-year landmark analysis comparing participants with and without DR diagnosed prior to the landmark.

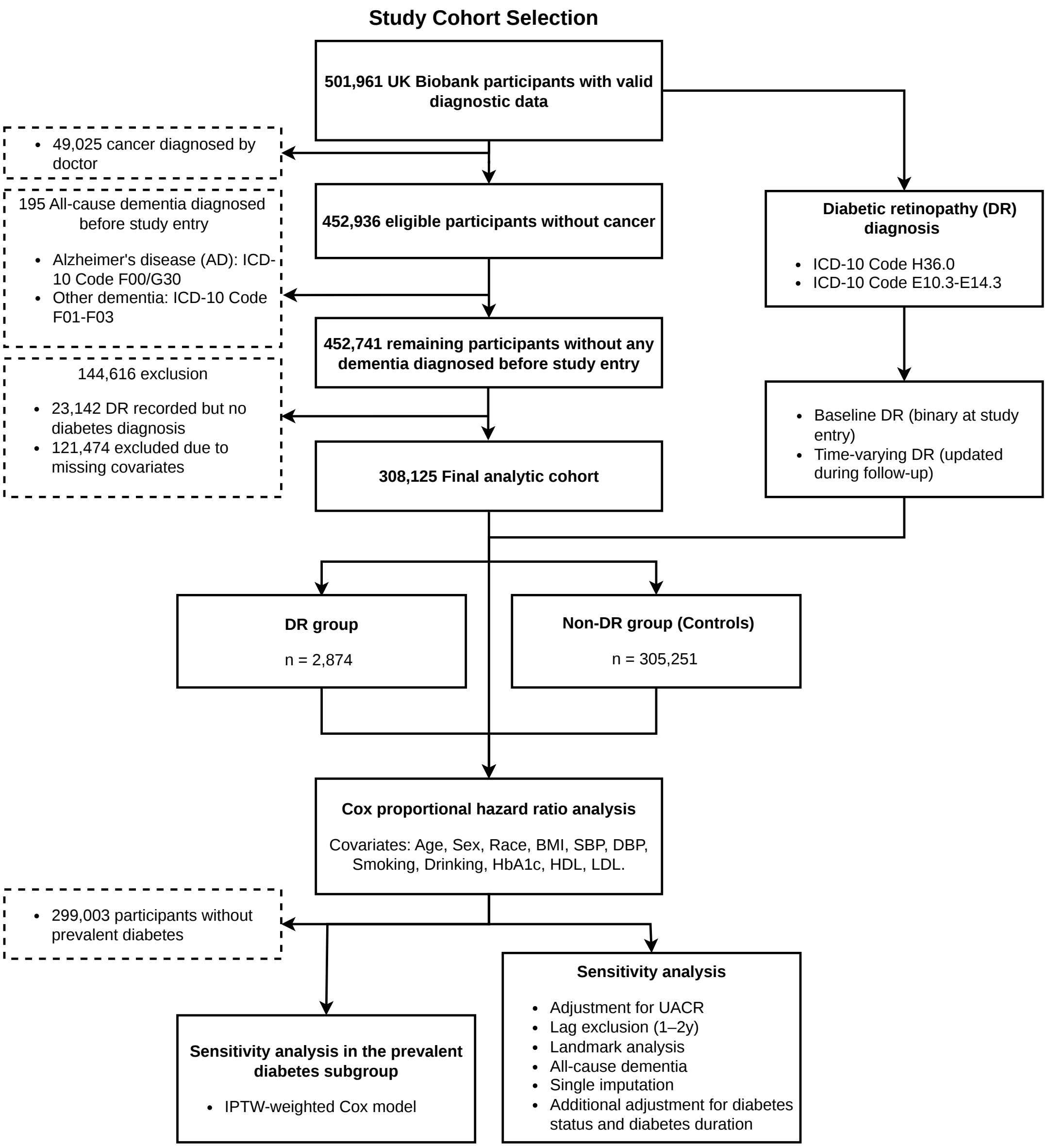

Figure S1. Flowchart of study cohort selection.

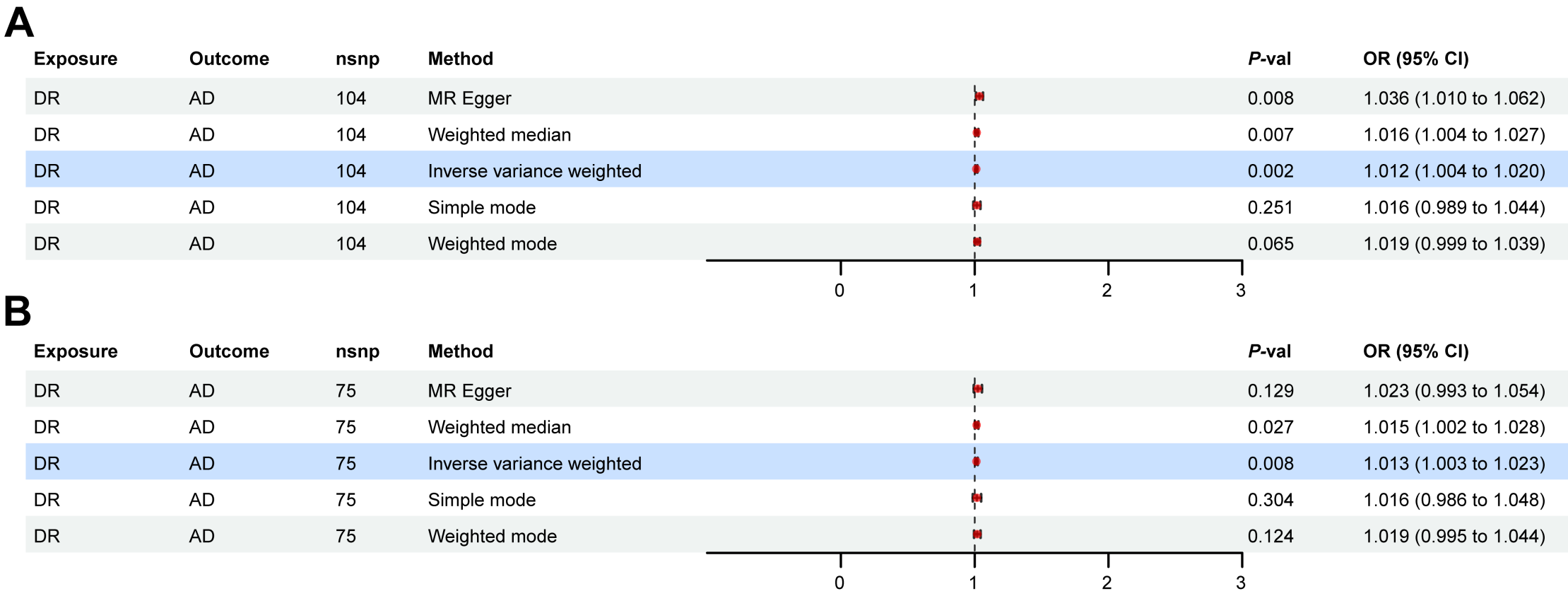

A

Exposure	Outcome	nsnp	Method	*P*-val	OR (95% CI)
DR	AD	104	MR Egger	0.008	1.036 (1.010 to 1.062)
DR	AD	104	Weighted median	0.007	1.016 (1.004 to 1.027)
DR	AD	104	Inverse variance weighted	0.002	1.012 (1.004 to 1.020)
DR	AD	104	Simple mode	0.251	1.016 (0.989 to 1.044)
DR	AD	104	Weighted mode	0.065	1.019 (0.999 to 1.039)

B

Exposure	Outcome	nsnp	Method	*P*-val	OR (95% CI)
DR	AD	75	MR Egger	0.129	1.023 (0.993 to 1.054)
DR	AD	75	Weighted median	0.027	1.015 (1.002 to 1.028)
DR	AD	75	Inverse variance weighted	0.008	1.013 (1.003 to 1.023)
DR	AD	75	Simple mode	0.304	1.016 (0.986 to 1.048)
DR	AD	75	Weighted mode	0.124	1.019 (0.995 to 1.044)

Figure S2. Causal effect of DR on AD as estimated by MR analysis. **Panel A.** MR results of European populations. **Panel B.** MR results of multi-populations.

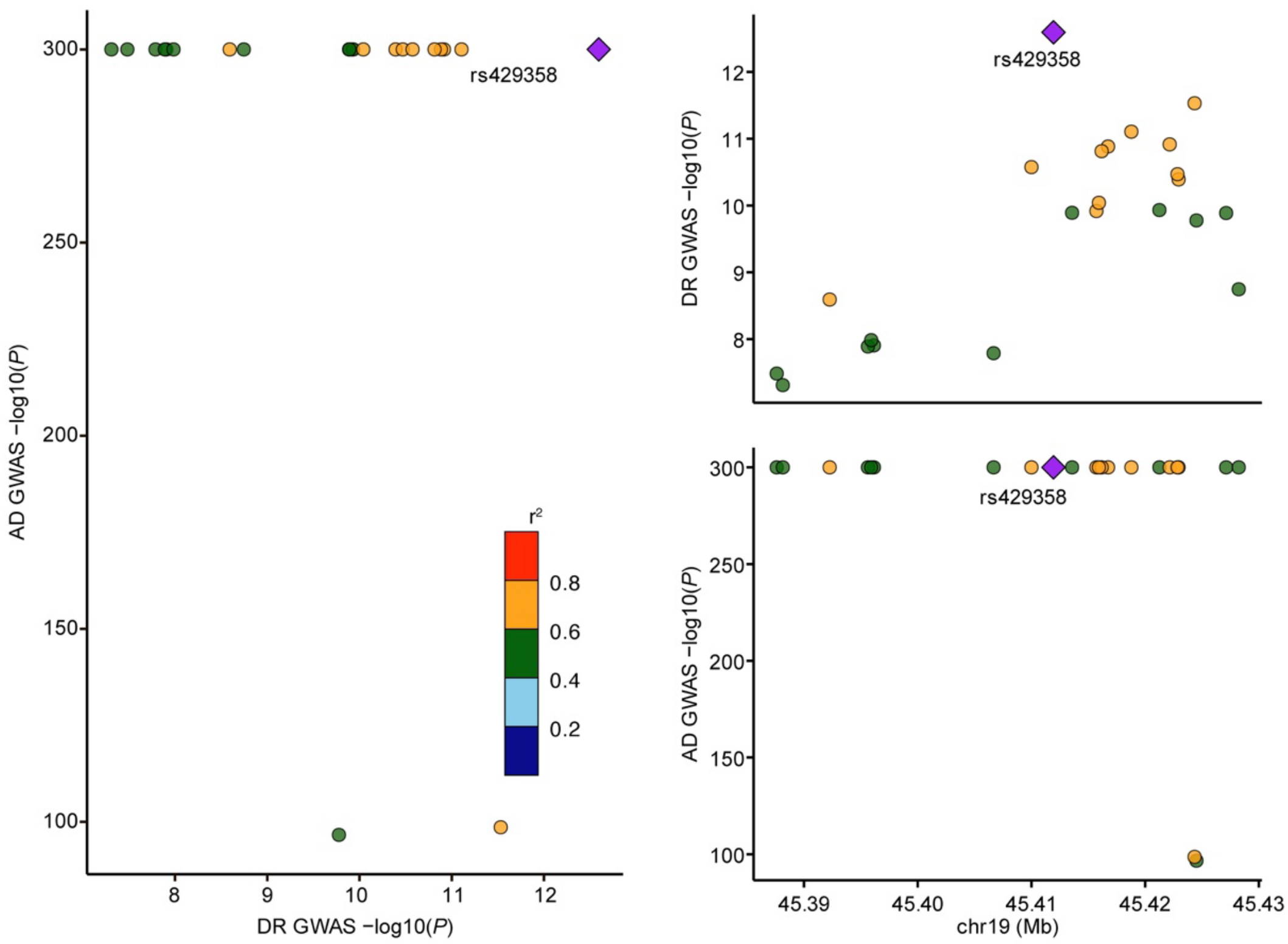

Figure S3. Locus-level comparison of association signals between DR and AD.

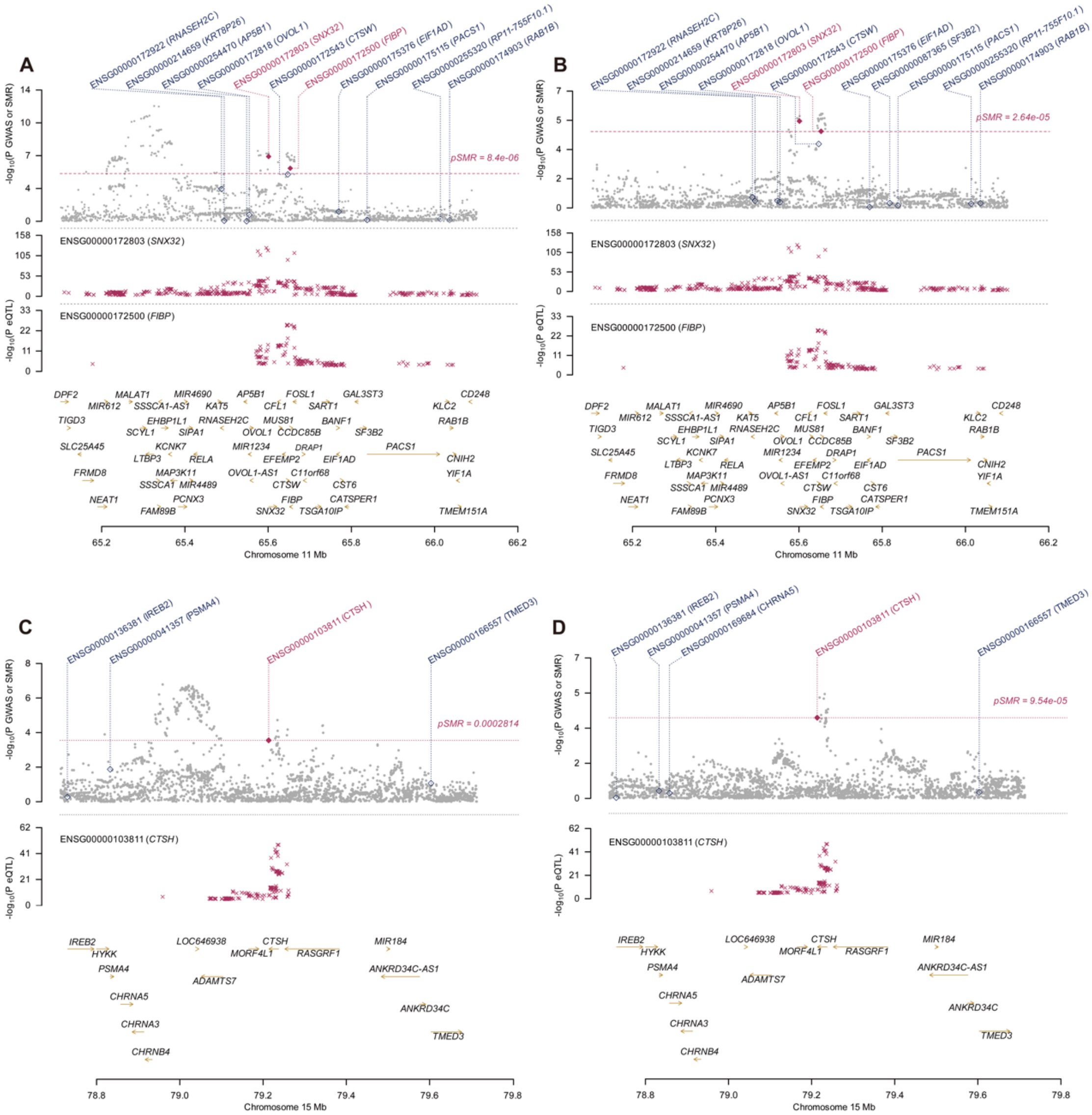

Figure S4. SMR locus plots for representative shared genes between DR and AD. **Panel A.** PsychENCODE Consortium eQTL–DR SMR results. **Panel B.** PsychENCODE Consortium eQTL-AD SMR results. **Panel C.** GTEx Consortium v8 whole blood eQTL-DR SMR results. **Panel D.** GTEx Consortium v8 whole blood eQTL-AD SMR results.

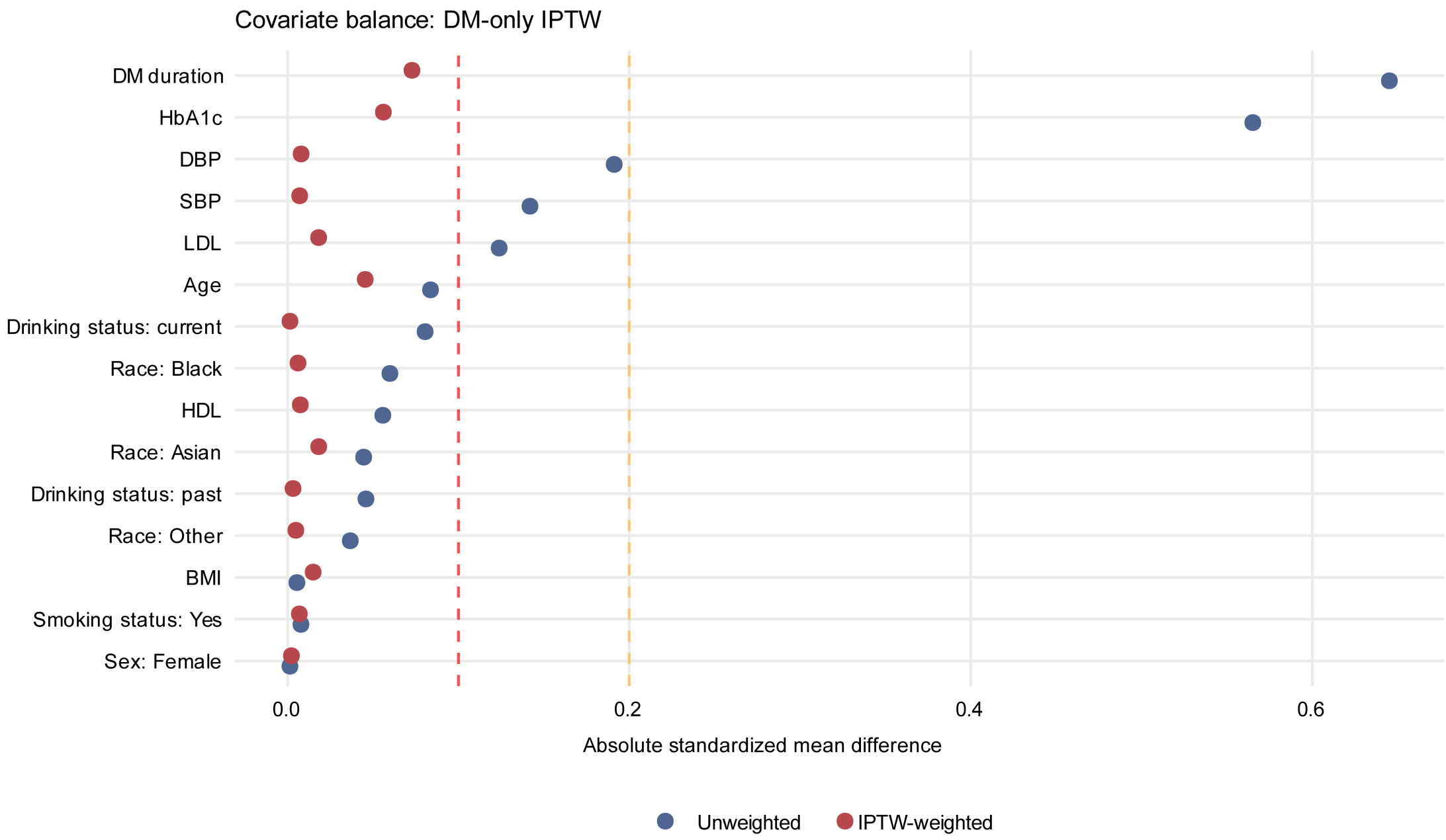

Figure S5. Love plot showing covariate balance before and after inverse probability of treatment weighting in the diabetes-restricted cohort. Absolute SMDs for all covariates included in the propensity score model are presented. The dashed red line indicates the threshold of SMD = 0.1.

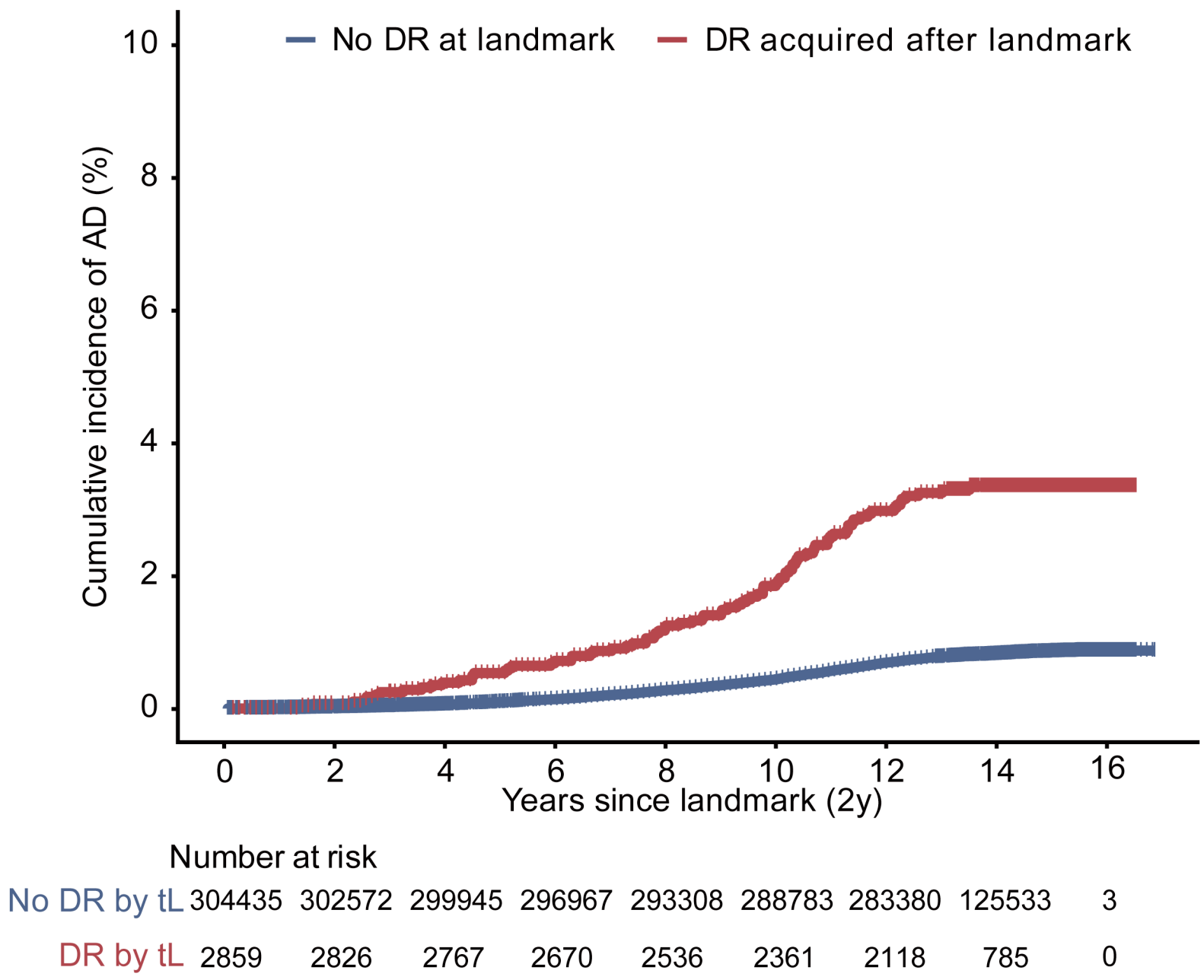

Figure S6. Cumulative incidence curves of AD from the 2-year landmark analysis comparing participants with and without DR diagnosed prior to the landmark.

Supplementary tables:

Table S1. Summary of GWAS datasets used in this study.
Table S2. Instrumental variable table.
Table S3. Instrumental variable table (without APOE).
Table S4. Sensitivity validation of MR (without APOE).
Table S5. Results of SMR analyses.
Table S6. MR estimates for the associations between DR, candidate mediators, and AD.
Table S7. Mediating role of each mediator in the causal associations between DR and AD.
Table S8. Baseline Cox regression analysis.
Table S9. Standardized mean differences of baseline covariates before and after inverse probability of treatment weighting in the diabetes-restricted cohort.
Table S10. Sensitivity analysis.
Table S11. Landmark diagnostics.
Table S12. Lag exclusion analysis.

Table S1. Summary of GWAS datasets used in this study.

Name	ID	Sample size	Database
DR	GCST90475689	29,668 European ancestry cases, 402,541 European ancestry controls	GWAS Catalog
Multi-population DR	GCST90479891	11,781 African American or Afro-Caribbean cases, 103,431 African American or Afro-Caribbean controls, 5,658 Hispanic or Latin American cases, 51,648 Hispanic or Latin American controls, 483 East Asian ancestry cases, 6,078 East Asian ancestry controls, 29,668 European ancestry cases, 402,541 European ancestry controls	GWAS Catalog
AD	GCST007320	24,087 European ancestry late-onset Alzheimer's disease cases, 47,793 European ancestry individuals with family history of Alzheimer's disease, 383,378 European ancestry controls	GWAS Catalog
HDL cholesterol	ieu-b-109	403,943	IEU OpenGWAS
LDL cholesterol	ieu-b-110	440,546	IEU OpenGWAS
Triglycerides	ieu-b-111	441,016	IEU OpenGWAS
Adiponectin	ieu-a-1	39,883	IEU OpenGWAS
Saturated fatty acids	met-d-SFA	114,999	IEU OpenGWAS
Polyunsaturated fatty acids	met-d-PUFA	114,999	IEU OpenGWAS
Omega-6 fatty acids	met-d-Omega_6	114,999	IEU OpenGWAS
Omega-3 fatty acids	met-d-Omega_3	114,999	IEU OpenGWAS
Monounsaturated fatty acids	met-d-MUFA	114,999	IEU OpenGWAS
Glutamate	met-a-466	7,804	IEU OpenGWAS
Body mass index	ukb-b-19953	461,460	IEU OpenGWAS
Proinsulin levels	ebi-a-GCST001212	10,701	IEU OpenGWAS
Fasting blood glucose	ebi-a-GCST000568	46,186	IEU OpenGWAS
Apolipoprotein B	ieu-b-108	439,214	IEU OpenGWAS
HbA1c	ebi-a-GCST90014006	389,889	IEU OpenGWAS

Table S2. Instrumental variable table.

SNP	Effect allele	Other allele	N	EAF	*β*	SE	*P*- value	R²	F-statistic
rs60069508	G	C	432209	0.789	-0.077	0.011	6.74E-13	1.19E-04	51.620
rs12121576	A	G	432209	0.823	0.063	0.011	2.58E-08	7.17E-05	31.002
rs7531118	T	C	432209	0.501	-0.053	0.009	9.72E-10	8.65E-05	37.381
rs66464442	C	A	432209	0.694	-0.048	0.009	1.55E-08	7.40E-05	31.994
rs1493694	C	T	432209	0.899	-0.074	0.013	2.27E-08	7.23E-05	31.245
rs9787073	T	C	432209	0.590	-0.057	0.009	5.13E-11	9.98E-05	43.126
rs12119128	G	A	432209	0.725	-0.055	0.009	1.65E-09	8.41E-05	36.345
rs17406570	G	C	432209	0.918	0.084	0.015	3.12E-08	7.09E-05	30.633
rs340874	T	C	432209	0.450	-0.056	0.008	5.41E-12	1.10E-04	47.533
rs348330	G	A	432209	0.398	0.060	0.009	1.46E-11	1.05E-04	45.586
rs1260326	T	C	432209	0.414	-0.062	0.008	1.43E-13	1.26E-04	54.668
rs67994640	A	G	432209	0.419	0.045	0.008	2.77E-08	7.14E-05	30.864
rs17030980	T	C	432209	0.881	0.140	0.013	1.04E-25	2.54E-04	109.891
rs151141151	G	A	432209	0.835	-0.066	0.011	2.88E-09	8.16E-05	35.263
rs7592940	T	C	432209	0.464	0.055	0.008	1.62E-11	1.05E-04	45.389
rs243083	A	G	432209	0.546	-0.062	0.008	1.19E-14	1.38E-04	59.562
rs11688682	G	C	432209	0.765	0.088	0.012	3.48E-14	1.33E-04	57.445
rs17410535	T	C	432209	0.719	-0.052	0.009	7.64E-09	7.72E-05	33.364
rs10195252	T	C	432209	0.595	0.077	0.008	2.17E-21	2.09E-04	90.187
rs2140046	T	C	432209	0.645	-0.051	0.009	9.76E-09	7.61E-05	32.888
rs2713549	A	G	432209	0.366	-0.087	0.009	2.53E-24	2.40E-04	103.557
rs11712037	C	G	432209	0.879	0.115	0.013	8.82E-19	1.81E-04	78.307
rs35352848	T	C	432209	0.800	0.072	0.010	1.69E-12	1.15E-04	49.816
rs704366	A	G	432209	0.152	-0.071	0.012	5.34E-09	7.88E-05	34.062
rs6551302	A	G	432209	0.209	-0.060	0.011	3.00E-08	7.10E-05	30.710
rs7613951	C	T	432209	0.771	0.059	0.009	4.18E-10	9.03E-05	39.029
rs9869577	C	T	432209	0.503	0.050	0.008	1.88E-09	8.35E-05	36.094
rs7651090	A	G	432209	0.685	-0.109	0.008	4.65E-38	3.85E-04	166.344
rs6777684	A	G	432209	0.396	-0.067	0.009	1.01E-14	1.39E-04	59.874
rs1801214	C	T	432209	0.397	-0.102	0.008	6.52E-35	3.51E-04	151.944
rs7692088	G	C	432209	0.480	-0.048	0.008	6.29E-09	7.81E-05	33.742
rs10938397	A	G	432209	0.568	-0.056	0.008	3.24E-12	1.12E-04	48.536
rs372533566	T	A	432209	0.459	0.082	0.014	3.66E-09	8.05E-05	34.799
rs151411	A	C	432209	0.804	-0.065	0.012	4.02E-08	6.97E-05	30.140
rs11727676	T	C	432209	0.906	-0.080	0.013	2.70E-09	8.19E-05	35.390
rs6837634	G	A	432209	0.720	0.067	0.010	2.48E-12	1.14E-04	49.063
rs6552825	T	A	432209	0.819	0.070	0.010	2.41E-11	1.03E-04	44.608
rs6889788	G	A	432209	0.818	0.068	0.011	9.12E-10	8.68E-05	37.505
rs11748742	C	T	432209	0.556	-0.045	0.008	3.98E-08	6.98E-05	30.160
rs173964	A	G	432209	0.266	-0.079	0.010	2.58E-16	1.55E-04	67.103
rs76160968	A	G	432209	0.953	-0.164	0.018	2.77E-20	1.97E-04	85.146
rs1650507	G	A	432209	0.784	-0.053	0.010	4.32E-08	6.94E-05	30.001
rs2714337	A	T	432209	0.666	-0.070	0.009	2.28E-15	1.45E-04	62.811
rs35261542	C	A	432209	0.736	-0.109	0.009	4.68E-34	3.42E-04	148.025
rs2394893	G	A	432209	0.397	-0.056	0.009	1.14E-09	8.57E-05	37.064
rs9266640	G	T	432209	0.816	-0.062	0.011	5.00E-09	7.91E-05	34.189
rs9366803	T	C	432209	0.949	-0.208	0.022	3.52E-21	2.06E-04	89.229
rs9275532	C	G	432209	0.895	-0.176	0.013	3.31E-43	4.39E-04	189.921
rs9462935	C	T	432209	0.768	-0.075	0.010	3.66E-15	1.43E-04	61.873
rs11759026	A	G	432209	0.772	-0.073	0.010	4.82E-14	1.31E-04	56.801
rs9986829	G	A	432209	0.493	0.055	0.008	6.92E-11	9.84E-05	42.542
rs1635852	T	C	432209	0.498	0.080	0.008	6.86E-24	2.35E-04	101.580
rs7801117	G	A	432209	0.696	-0.053	0.009	4.07E-09	8.00E-05	34.589
rs1182435	G	C	432209	0.361	-0.066	0.008	8.48E-15	1.39E-04	60.220
rs73663955	C	A	432209	0.611	-0.049	0.009	1.78E-08	7.34E-05	31.716
rs12550613	G	C	432209	0.432	-0.061	0.008	1.99E-14	1.35E-04	58.543

rs3808447	G	A	432209	0.495	0.069	0.009	1.01E-15	1.49E-04	64.403
rs13266634	C	T	432209	0.697	0.092	0.009	1.21E-25	2.53E-04	109.581
rs10811661	T	C	432209	0.825	0.154	0.011	4.10E-44	4.49E-04	194.077
rs10972500	G	A	432209	0.836	0.063	0.011	1.76E-08	7.34E-05	31.745
rs2796441	G	A	432209	0.591	0.054	0.008	2.33E-11	1.03E-04	44.672
rs7861905	G	C	432209	0.934	-0.104	0.016	3.22E-11	1.02E-04	44.036
rs115478735	A	T	432209	0.808	-0.062	0.010	8.66E-10	8.70E-05	37.605
rs376993806	A	G	432209	0.294	-0.067	0.009	2.25E-12	1.14E-04	49.249
rs1317548	G	A	432209	0.809	-0.084	0.010	9.14E-16	1.49E-04	64.607
rs2812536	G	C	432209	0.418	-0.052	0.009	5.72E-09	7.85E-05	33.927
rs11001118	G	A	432209	0.961	-0.115	0.021	2.98E-08	7.11E-05	30.719
rs810517	C	T	432209	0.549	0.076	0.008	1.37E-20	2.00E-04	86.533
rs10882104	T	C	432209	0.630	0.103	0.008	2.47E-34	3.45E-04	149.299
rs2251772	T	C	432209	0.726	0.055	0.009	2.37E-09	8.25E-05	35.640
rs57543781	T	G	432209	0.458	-0.072	0.009	1.17E-15	1.48E-04	64.116
rs4929965	A	G	432209	0.384	0.056	0.008	5.49E-12	1.10E-04	47.505
rs234864	G	A	432209	0.460	0.088	0.008	2.94E-27	2.71E-04	116.955
rs7124355	A	G	432209	0.341	0.071	0.009	4.82E-17	1.63E-04	70.407
rs577964	T	G	432209	0.687	0.049	0.009	4.45E-08	6.93E-05	29.943
rs2293576	G	A	432209	0.668	0.055	0.008	5.69E-11	9.93E-05	42.925
rs12789028	G	A	432209	0.813	-0.072	0.010	5.12E-13	1.21E-04	52.159
rs9667947	T	C	432209	0.841	0.077	0.011	1.51E-11	1.05E-04	45.516
rs12582638	G	A	432209	0.502	0.051	0.009	1.05E-08	7.58E-05	32.748
rs11063069	A	G	432209	0.788	-0.059	0.010	7.81E-10	8.75E-05	37.807
rs76895963	T	G	432209	0.982	0.389	0.043	3.86E-19	1.85E-04	79.939
rs4963661	C	T	432209	0.267	0.051	0.009	2.54E-08	7.18E-05	31.029
rs10842987	A	C	432209	0.796	0.077	0.010	6.05E-14	1.30E-04	56.355
rs111164090	A	G	432209	0.599	-0.048	0.009	2.45E-08	7.20E-05	31.102
rs1351394	T	C	432209	0.484	-0.062	0.008	5.96E-15	1.41E-04	60.915
rs28644184	T	C	432209	0.472	-0.057	0.009	3.46E-11	1.02E-04	43.900
rs34584161	A	G	432209	0.760	0.060	0.009	9.54E-11	9.70E-05	41.914
rs1327315	C	T	432209	0.722	0.072	0.009	1.77E-15	1.46E-04	63.305
rs1414319	G	A	432209	0.424	0.051	0.008	1.56E-09	8.43E-05	36.455
rs12867262	A	G	432209	0.677	0.053	0.009	4.21E-09	7.99E-05	34.526
rs2370982	C	T	432209	0.793	-0.074	0.010	3.27E-14	1.33E-04	57.567
rs11634182	T	C	432209	0.513	-0.050	0.008	3.96E-09	8.02E-05	34.646
rs113139584	C	T	432209	0.902	-0.084	0.014	1.02E-09	8.62E-05	37.278
rs4775467	G	A	432209	0.589	0.047	0.008	3.42E-09	8.08E-05	34.931
rs34784690	C	T	432209	0.308	-0.059	0.009	7.40E-11	9.81E-05	42.411
rs3759831	C	G	432209	0.268	0.077	0.009	3.83E-18	1.74E-04	75.407
rs3874658	T	C	432209	0.861	0.076	0.013	7.93E-09	7.70E-05	33.293
rs1421085	T	C	432209	0.593	-0.145	0.008	8.25E-74	7.64E-04	330.304
rs72804106	G	T	432209	0.899	0.084	0.014	4.68E-10	8.98E-05	38.808
rs12603448	A	G	432209	0.814	-0.065	0.011	1.75E-09	8.38E-05	36.237
rs8076462	G	C	432209	0.276	0.051	0.009	4.84E-08	6.89E-05	29.779
rs11652684	T	G	432209	0.810	0.073	0.011	3.07E-12	1.13E-04	48.646
rs59606758	G	A	432209	0.680	0.052	0.009	1.62E-09	8.42E-05	36.382
rs13381608	C	T	432209	0.803	-0.062	0.010	9.40E-10	8.66E-05	37.446
rs571312	C	A	432209	0.765	-0.065	0.009	6.88E-14	1.30E-04	56.102
rs2115107	G	A	432209	0.612	-0.047	0.008	1.25E-08	7.50E-05	32.411
rs11881199	T	C	432209	0.506	0.044	0.008	2.95E-08	7.11E-05	30.741
rs58489806	C	T	432209	0.910	-0.102	0.014	2.22E-13	1.24E-04	53.802
rs429358	T	C	432209	0.860	0.085	0.012	2.55E-13	1.24E-04	53.527
rs35226705	A	C	432209	0.535	-0.049	0.008	1.63E-09	8.41E-05	36.369
rs1232605	A	G	432209	0.584	-0.047	0.008	1.31E-08	7.48E-05	32.318
rs6072223	T	A	432209	0.844	-0.072	0.011	8.25E-11	9.76E-05	42.198
rs3747207	G	A	432209	0.781	-0.091	0.009	7.15E-22	2.14E-04	92.379

Table S3. Instrumental variable table (without APOE).

SNP	Effect allele	Other allele	N	EAF	*β*	SE	*P*- value	R²	F-statistic
rs1493694	C	T	432209	0.899	-0.074	0.013	2.27E-08	7.23E-05	31.245
rs9787073	T	C	432209	0.590	-0.057	0.009	5.13E-11	9.98E-05	43.126
rs348330	G	A	432209	0.398	0.060	0.009	1.46E-11	1.05E-04	45.586
rs67994640	A	G	432209	0.419	0.045	0.008	2.77E-08	7.14E-05	30.864
rs17030980	T	C	432209	0.881	0.140	0.013	1.04E-25	2.54E-04	109.891
rs11688682	G	C	432209	0.765	0.088	0.012	3.48E-14	1.33E-04	57.445
rs17410535	T	C	432209	0.719	-0.052	0.009	7.64E-09	7.72E-05	33.364
rs10195252	T	C	432209	0.595	0.077	0.008	2.17E-21	2.09E-04	90.187
rs2140046	T	C	432209	0.645	-0.051	0.009	9.76E-09	7.61E-05	32.888
rs2713549	A	G	432209	0.366	-0.087	0.009	2.53E-24	2.40E-04	103.557
rs11712037	C	G	432209	0.879	0.115	0.013	8.82E-19	1.81E-04	78.307
rs35352848	T	C	432209	0.800	0.072	0.010	1.69E-12	1.15E-04	49.816
rs6551302	A	G	432209	0.209	-0.060	0.011	3.00E-08	7.10E-05	30.710
rs7613951	C	T	432209	0.771	0.059	0.009	4.18E-10	9.03E-05	39.029
rs7651090	A	G	432209	0.685	-0.109	0.008	4.65E-38	3.85E-04	166.344
rs6777684	A	G	432209	0.396	-0.067	0.009	1.01E-14	1.39E-04	59.874
rs1801214	C	T	432209	0.397	-0.102	0.008	6.52E-35	3.51E-04	151.944
rs10938397	A	G	432209	0.568	-0.056	0.008	3.24E-12	1.12E-04	48.536
rs11727676	T	C	432209	0.906	-0.080	0.013	2.70E-09	8.19E-05	35.390
rs6837634	G	A	432209	0.720	0.067	0.010	2.48E-12	1.14E-04	49.063
rs6552825	T	A	432209	0.819	0.070	0.010	2.41E-11	1.03E-04	44.608
rs6889788	G	A	432209	0.818	0.068	0.011	9.12E-10	8.68E-05	37.505
rs11748742	C	T	432209	0.556	-0.045	0.008	3.98E-08	6.98E-05	30.160
rs173964	A	G	432209	0.266	-0.079	0.010	2.58E-16	1.55E-04	67.103
rs1650507	G	A	432209	0.784	-0.053	0.010	4.32E-08	6.94E-05	30.001
rs2714337	A	T	432209	0.666	-0.070	0.009	2.28E-15	1.45E-04	62.811
rs35261542	C	A	432209	0.736	-0.109	0.009	4.68E-34	3.42E-04	148.025
rs9266640	G	T	432209	0.816	-0.062	0.011	5.00E-09	7.91E-05	34.189
rs9462935	C	T	432209	0.768	-0.075	0.010	3.66E-15	1.43E-04	61.873
rs9986829	G	A	432209	0.493	0.055	0.008	6.92E-11	9.84E-05	42.542
rs1182435	G	C	432209	0.361	-0.066	0.008	8.48E-15	1.39E-04	60.220
rs73663955	C	A	432209	0.611	-0.049	0.009	1.78E-08	7.34E-05	31.716
rs12550613	G	C	432209	0.432	-0.061	0.008	1.99E-14	1.35E-04	58.543
rs3808447	G	A	432209	0.495	0.069	0.009	1.01E-15	1.49E-04	64.403
rs13266634	C	T	432209	0.697	0.092	0.009	1.21E-25	2.53E-04	109.581
rs2796441	G	A	432209	0.591	0.054	0.008	2.33E-11	1.03E-04	44.672
rs7861905	G	C	432209	0.934	-0.104	0.016	3.22E-11	1.02E-04	44.036
rs115478735	A	T	432209	0.808	-0.062	0.010	8.66E-10	8.70E-05	37.605
rs1317548	G	A	432209	0.809	-0.084	0.010	9.14E-16	1.49E-04	64.607
rs2812536	G	C	432209	0.418	-0.052	0.009	5.72E-09	7.85E-05	33.927
rs11001118	G	A	432209	0.961	-0.115	0.021	2.98E-08	7.11E-05	30.719
rs810517	C	T	432209	0.549	0.076	0.008	1.37E-20	2.00E-04	86.533
rs10882104	T	C	432209	0.630	0.103	0.008	2.47E-34	3.45E-04	149.299
rs57543781	T	G	432209	0.458	-0.072	0.009	1.17E-15	1.48E-04	64.116
rs4929965	A	G	432209	0.384	0.056	0.008	5.49E-12	1.10E-04	47.505
rs234864	G	A	432209	0.460	0.088	0.008	2.94E-27	2.71E-04	116.955
rs7124355	A	G	432209	0.341	0.071	0.009	4.82E-17	1.63E-04	70.407
rs577964	T	G	432209	0.687	0.049	0.009	4.45E-08	6.93E-05	29.943
rs12582638	G	A	432209	0.502	0.051	0.009	1.05E-08	7.58E-05	32.748
rs11063069	A	G	432209	0.788	-0.059	0.010	7.81E-10	8.75E-05	37.807
rs4963661	C	T	432209	0.267	0.051	0.009	2.54E-08	7.18E-05	31.029
rs10842987	A	C	432209	0.796	0.077	0.010	6.05E-14	1.30E-04	56.355
rs111164090	A	G	432209	0.599	-0.048	0.009	2.45E-08	7.20E-05	31.102
rs1351394	T	C	432209	0.484	-0.062	0.008	5.96E-15	1.41E-04	60.915
rs34584161	A	G	432209	0.760	0.060	0.009	9.54E-11	9.70E-05	41.914
rs1327315	C	T	432209	0.722	0.072	0.009	1.77E-15	1.46E-04	63.305

rs1414319	G	A	432209	0.424	0.051	0.008	1.56E-09	8.43E-05	36.455
rs2370982	C	T	432209	0.793	-0.074	0.010	3.27E-14	1.33E-04	57.567
rs11634182	T	C	432209	0.513	-0.050	0.008	3.96E-09	8.02E-05	34.646
rs113139584	C	T	432209	0.902	-0.084	0.014	1.02E-09	8.62E-05	37.278
rs4775467	G	A	432209	0.589	0.047	0.008	3.42E-09	8.08E-05	34.931
rs34784690	C	T	432209	0.308	-0.059	0.009	7.40E-11	9.81E-05	42.411
rs3759831	C	G	432209	0.268	0.077	0.009	3.83E-18	1.74E-04	75.407
rs3874658	T	C	432209	0.861	0.076	0.013	7.93E-09	7.70E-05	33.293
rs1421085	T	C	432209	0.593	-0.145	0.008	8.25E-74	7.64E-04	330.304
rs72804106	G	T	432209	0.899	0.084	0.014	4.68E-10	8.98E-05	38.808
rs59606758	G	A	432209	0.680	0.052	0.009	1.62E-09	8.42E-05	36.382
rs571312	C	A	432209	0.765	-0.065	0.009	6.88E-14	1.30E-04	56.102
rs2115107	G	A	432209	0.612	-0.047	0.008	1.25E-08	7.50E-05	32.411
rs11881199	T	C	432209	0.506	0.044	0.008	2.95E-08	7.11E-05	30.741
rs1232605	A	G	432209	0.584	-0.047	0.008	1.31E-08	7.48E-05	32.318
rs3747207	G	A	432209	0.781	-0.091	0.009	7.15E-22	2.14E-04	92.379

Table S4. Sensitivity validation of MR (without APOE).

Exposure	Outcome	Cochran's Q test		MR-Egger intercept test		MR-PRESSO
		Q statistic	*P*- value	Intercept	*P*- value	*P*- value of global test
DR(without APOE)	AD	77.573	0.250	-0.001	0.293	0.254

Table S5. Results of SMR analyses.

eQTL data	Trait	ProbeID	Gene	TopSNP	A1	A2	EAF	*β* GWAS	*P* GWAS	*β* eQTL	*P* eQTL	*β* SMR	*P* SMR	*P* HEIDI	*P* FDR
PsychENCODE Consortium	AD	ENSG00000203710	*CR1*	rs679515	C	T	0.83	-0.03	6.83E-19	-0.49	1.53E-45	0.05	5.39E-14	0.24	2.67E-10
PsychENCODE Consortium	AD	ENSG00000168077	*SCARA3*	rs9331908	T	C	0.35	0.01	5.55E-11	-0.09	2.69E-08	-0.17	2.23E-05	0.07	1.58E-02
PsychENCODE Consortium	AD	ENSG00000172803	*SNX32*	rs111953392	A	G	0.16	-0.01	4.22E-06	0.40	2.34E-126	-0.03	6.26E-06	0.22	6.90E-03
PsychENCODE Consortium	AD	ENSG00000172500	*FIBP*	rs1204650	C	A	0.81	0.01	4.41E-06	-0.14	5.25E-26	-0.09	2.56E-05	0.20	1.69E-02
PsychENCODE Consortium	AD	ENSG00000178226	*PRSS36*	rs55766044	T	C	0.28	-0.01	7.60E-08	0.19	4.13E-12	-0.07	2.15E-05	0.43	1.58E-02
PsychENCODE Consortium	AD	ENSG00000167840	*ZNF232*	rs55922100	T	C	0.11	0.02	6.15E-08	-0.38	1.00E-30	-0.05	9.56E-07	0.11	1.36E-03
PsychENCODE Consortium	AD	ENSG00000159640	*ACE*	rs6504163	T	C	0.64	0.01	8.86E-07	-0.14	3.44E-11	-0.08	7.89E-05	0.18	4.12E-02
PsychENCODE Consortium	AD	ENSG00000087589	*CASS4*	rs718022	A	G	0.08	-0.02	1.44E-08	0.23	1.06E-10	-0.10	2.04E-05	0.15	1.58E-02
PsychENCODE Consortium	DR	ENSG00000162413	*KLHL21*	rs7513688	A	G	0.37	0.03	3.05E-05	-0.08	4.00E-09	-0.46	6.68E-04	0.07	4.60E-02
PsychENCODE Consortium	DR	ENSG00000197245	*FAM110D*	rs72666338	T	A	0.31	0.04	1.04E-05	0.22	2.87E-18	0.18	8.36E-05	0.09	1.26E-02
PsychENCODE Consortium	DR	ENSG00000227207	*RPL31P12*	rs2568960	G	A	0.64	0.05	6.79E-09	-0.68	7.02E-97	-0.07	2.34E-08	0.11	4.59E-05
PsychENCODE Consortium	DR	ENSG00000242252	*BGLAP*	rs2075165	A	G	0.46	0.04	8.10E-06	-0.19	2.91E-19	-0.19	6.45E-05	0.36	1.09E-02
PsychENCODE Consortium	DR	ENSG00000198952	*SMG5*	rs7542993	C	A	0.46	0.04	2.13E-05	-0.07	1.79E-13	-0.50	2.32E-04	0.59	2.45E-02
PsychENCODE Consortium	DR	ENSG00000198468	*FLVCR1-AS1*	rs10779588	C	G	0.53	0.03	6.47E-04	0.86	7.25E-258	0.03	6.88E-04	0.07	4.60E-02
PsychENCODE Consortium	DR	ENSG00000162769	*FLVCR1*	rs41297446	T	C	0.19	-0.04	2.03E-05	0.15	2.05E-13	-0.29	2.27E-04	0.27	2.43E-02
PsychENCODE Consortium	DR	ENSG00000115486	*GGCX*	rs2366639	G	A	0.43	-0.03	6.11E-05	0.25	1.35E-32	-0.13	1.46E-04	0.30	1.84E-02
PsychENCODE Consortium	DR	ENSG00000152253	*SPC25*	rs7606215	T	A	0.09	-0.05	2.15E-04	-0.60	2.25E-40	0.08	3.64E-04	0.15	3.14E-02
PsychENCODE Consortium	DR	ENSG00000145029	*NICN1*	rs6798112	G	A	0.78	0.04	8.57E-05	-0.12	5.76E-14	-0.31	5.00E-04	0.53	3.84E-02
PsychENCODE Consortium	DR	ENSG00000242142	*SERBP1P3*	rs2581800	G	A	0.60	0.03	1.64E-04	0.34	8.08E-55	0.09	2.49E-04	0.73	2.53E-02
PsychENCODE Consortium	DR	ENSG00000272305	*RP11-894J14.5*	rs1864367	C	T	0.60	0.03	2.29E-04	0.31	1.44E-26	0.10	4.96E-04	0.50	3.84E-02
PsychENCODE Consortium	DR	ENSG00000168374	*ARF4*	rs10049375	A	G	0.15	0.05	1.39E-05	0.12	6.31E-18	0.38	1.04E-04	0.38	1.48E-02
PsychENCODE Consortium	DR	ENSG00000175105	*ZNF654*	rs7637431	C	T	0.85	0.06	7.54E-06	-0.06	2.34E-11	-0.87	1.99E-04	0.41	2.25E-02
PsychENCODE Consortium	DR	ENSG00000179021	*C3orf38*	rs6551293	T	C	0.84	0.05	3.52E-05	-0.08	6.21E-13	-0.60	3.35E-04	0.32	2.98E-02
PsychENCODE Consortium	DR	ENSG00000169087	*HSPBAP1*	rs34876089	C	T	0.14	-0.04	2.20E-04	-0.24	1.68E-35	0.17	3.98E-04	0.06	3.31E-02
PsychENCODE Consortium	DR	ENSG00000171084	*FAM86JP*	rs13065725	A	C	0.17	-0.04	3.41E-04	0.86	1.30E-155	-0.05	3.86E-04	0.19	3.27E-02
PsychENCODE Consortium	DR	ENSG00000181322	*NME9*	rs7637162	G	A	0.74	-0.04	1.10E-05	0.27	6.40E-30	-0.14	4.14E-05	0.28	7.90E-03
PsychENCODE Consortium	DR	ENSG00000158220	*ESYT3*	rs1109088	A	G	0.57	-0.03	3.08E-05	-0.11	2.59E-18	0.31	1.69E-04	0.13	1.97E-02
PsychENCODE Consortium	DR	ENSG00000203632	*AC007690.1*	rs28366036	C	A	0.36	-0.04	3.58E-05	-0.13	1.61E-16	0.28	2.20E-04	0.58	2.42E-02
PsychENCODE Consortium	DR	ENSG00000109501	*WFS1*	rs9457	C	G	0.59	0.10	9.69E-34	0.12	1.45E-29	0.86	1.49E-16	0.16	1.46E-12
PsychENCODE Consortium	DR	ENSG00000145284	*SCD5*	rs7670515	T	A	0.65	-0.03	1.09E-04	-0.16	3.05E-31	0.20	2.42E-04	0.21	2.48E-02
PsychENCODE Consortium	DR	ENSG00000152583	*SPARCL1*	rs57201082	T	C	0.45	-0.03	1.52E-04	-0.07	4.72E-14	0.47	7.11E-04	0.63	4.69E-02
PsychENCODE Consortium	DR	ENSG00000246560	*RP11-10L12.4*	rs223341	A	T	0.47	-0.03	2.82E-04	-0.36	5.18E-40	0.09	4.61E-04	0.25	3.66E-02
PsychENCODE Consortium	DR	ENSG00000145354	*CISD2*	rs10004342	G	A	0.49	-0.03	1.10E-04	-0.07	1.65E-12	0.44	6.93E-04	0.81	4.60E-02
PsychENCODE Consortium	DR	ENSG00000164037	*SLC9B1*	rs4699039	A	C	0.49	-0.03	6.11E-05	-0.24	2.26E-31	0.14	1.50E-04	0.88	1.87E-02
PsychENCODE Consortium	DR	ENSG00000113594	*LIFR*	rs327287	T	G	0.53	0.04	3.82E-07	0.06	2.00E-20	0.64	8.48E-06	0.50	2.98E-03
PsychENCODE Consortium	DR	ENSG00000272335	*RP11-53O19.3*	rs7736952	T	C	0.37	0.04	1.81E-05	-0.16	3.92E-43	-0.23	4.26E-05	0.05	7.90E-03

PsychENCODE Consortium	DR	ENSG00000170571	*EMB*	rs10076490	C	A	0.44	0.04	9.49E-06	0.19	2.10E-67	0.19	1.78E-05	0.70	4.49E-03
PsychENCODE Consortium	DR	ENSG00000132837	*DMGDH*	rs10052346	T	G	0.37	-0.03	4.55E-05	0.21	4.60E-18	-0.16	2.25E-04	0.34	2.43E-02
PsychENCODE Consortium	DR	ENSG00000250378	*RP11-119J18.1*	rs4292443	T	C	0.34	0.03	2.04E-04	-0.28	6.99E-16	-0.11	7.42E-04	0.42	4.78E-02
PsychENCODE Consortium	DR	ENSG00000137414	*FAM8A1*	rs6937765	G	A	0.23	0.04	3.69E-05	0.08	6.27E-12	0.53	4.04E-04	0.06	3.31E-02
PsychENCODE Consortium	DR	ENSG00000047936	*ROS1*	rs9320602	C	T	0.14	-0.04	4.72E-04	0.26	5.41E-35	-0.15	7.69E-04	0.25	4.84E-02
PsychENCODE Consortium	DR	ENSG00000228408	*RP1-111D6.3*	rs9498250	C	T	0.30	0.03	1.01E-04	0.46	1.76E-107	0.07	1.29E-04	0.93	1.72E-02
PsychENCODE Consortium	DR	ENSG00000106123	*EPHB6*	rs10270914	T	C	0.06	0.07	1.81E-04	-0.14	2.89E-20	-0.47	5.22E-04	0.06	3.98E-02
PsychENCODE Consortium	DR	ENSG00000165125	*TRPV6*	rs10270914	T	C	0.06	0.07	1.81E-04	-0.16	1.54E-17	-0.42	6.08E-04	0.31	4.43E-02
PsychENCODE Consortium	DR	ENSG00000173295	*FAM86B3P*	rs2945253	C	T	0.49	0.05	9.55E-05	-0.61	1.86E-120	-0.08	1.19E-04	0.18	1.62E-02
PsychENCODE Consortium	DR	ENSG00000255457	*RP11-436P7.1*	rs1481907	C	G	0.13	0.05	6.82E-05	0.26	6.87E-14	0.18	4.37E-04	0.21	3.55E-02
PsychENCODE Consortium	DR	ENSG00000078674	*PCM1*	rs850835	G	C	0.62	0.04	4.09E-06	-0.12	1.31E-50	-0.34	1.07E-05	0.79	3.39E-03
PsychENCODE Consortium	DR	ENSG00000241852	*C8orf58*	rs13271626	G	C	0.33	0.04	1.72E-05	0.18	5.95E-17	0.21	1.32E-04	0.85	1.72E-02
PsychENCODE Consortium	DR	ENSG00000133863	*TEX15*	rs2543615	C	T	0.55	-0.04	4.95E-06	-0.09	2.71E-11	0.41	1.65E-04	0.35	1.96E-02
PsychENCODE Consortium	DR	ENSG00000029534	*ANK1*	rs3802315	T	G	0.24	-0.07	2.64E-12	-0.11	3.19E-08	0.63	1.43E-05	0.11	4.03E-03
PsychENCODE Consortium	DR	ENSG00000253596	*CTD-2320G14.2*	rs7846006	G	A	0.45	-0.03	1.46E-04	-0.38	2.97E-35	0.09	2.82E-04	0.54	2.74E-02
PsychENCODE Consortium	DR	ENSG00000231990	*RP11-53B5.1*	rs36110289	T	G	0.14	0.04	1.48E-04	0.29	1.47E-13	0.15	7.37E-04	0.10	4.78E-02
PsychENCODE Consortium	DR	ENSG00000011454	*RABGAP1*	rs12379325	G	T	0.12	-0.05	1.37E-05	-0.14	4.59E-19	0.40	9.27E-05	0.07	1.38E-02
PsychENCODE Consortium	DR	ENSG00000213221	*DNLZ*	rs3812553	T	G	0.04	0.09	7.57E-05	0.90	1.38E-81	0.10	1.06E-04	0.07	1.49E-02
PsychENCODE Consortium	DR	ENSG00000165626	*BEND7*	rs7918607	C	A	0.34	-0.04	2.81E-05	-0.21	1.80E-12	0.18	3.17E-04	0.13	2.91E-02
PsychENCODE Consortium	DR	ENSG00000075826	*SEC31B*	rs10509744	T	C	0.19	-0.04	1.06E-04	-0.18	4.36E-44	0.21	1.89E-04	0.57	2.16E-02
PsychENCODE Consortium	DR	ENSG00000203886	*CYP17A1-AS1*	rs284862	T	C	0.57	-0.03	4.90E-06	-0.20	1.10E-13	0.18	9.95E-05	0.16	1.44E-02
PsychENCODE Consortium	DR	ENSG00000148798	*INA*	rs11191641	T	C	0.48	-0.03	1.63E-04	0.11	3.68E-60	-0.29	2.38E-04	0.06	2.46E-02
PsychENCODE Consortium	DR	ENSG00000260461	*RP11-541N10.3*	rs35223321	T	C	0.54	0.04	5.46E-07	0.14	8.51E-22	0.28	8.97E-06	0.14	3.04E-03
PsychENCODE Consortium	DR	ENSG00000170959	*DCDC1*	rs7123466	C	T	0.29	0.04	2.60E-05	-0.11	1.61E-08	-0.35	7.42E-04	0.07	4.78E-02
PsychENCODE Consortium	DR	ENSG00000149485	*FADS1*	rs174544	A	C	0.30	-0.03	1.68E-04	-0.30	2.38E-67	0.11	2.35E-04	0.72	2.46E-02
PsychENCODE Consortium	DR	ENSG00000172803	*SNX32*	rs111953392	A	G	0.16	0.06	5.90E-08	0.40	2.34E-126	0.14	1.24E-07	0.07	1.52E-04
PsychENCODE Consortium	DR	ENSG00000172543	*CTSW*	rs596002	G	A	0.81	-0.05	1.21E-07	0.26	1.00E-15	-0.20	9.94E-06	0.49	3.26E-03
PsychENCODE Consortium	DR	ENSG00000172500	*FIBP*	rs1204650	C	A	0.81	-0.05	1.18E-07	-0.14	5.25E-26	0.38	2.21E-06	0.85	1.11E-03
PsychENCODE Consortium	DR	ENSG00000261070	*RP11-554A11.8*	rs72932540	G	A	0.12	-0.06	2.69E-05	0.37	1.18E-09	-0.15	5.50E-04	0.39	4.12E-02
PsychENCODE Consortium	DR	ENSG00000204381	*LAYN*	rs682239	T	C	0.59	-0.03	3.70E-04	0.24	7.67E-77	-0.12	4.71E-04	0.37	3.69E-02
PsychENCODE Consortium	DR	ENSG00000257815	*RP11-611E13.2*	rs79758480	C	T	0.16	0.04	4.46E-04	0.50	5.33E-46	0.08	6.52E-04	0.36	4.58E-02
PsychENCODE Consortium	DR	ENSG00000135100	*HNF1A*	rs2254971	C	G	0.40	0.03	1.30E-04	0.10	9.40E-22	0.31	3.81E-04	0.08	3.25E-02
PsychENCODE Consortium	DR	ENSG00000235423	*RP11-282O18.3*	rs10744149	A	T	0.77	0.04	6.87E-05	0.17	3.22E-11	0.24	6.41E-04	0.05	4.56E-02
PsychENCODE Consortium	DR	ENSG00000120697	*ALG5*	rs9576146	C	G	0.38	-0.04	4.12E-05	-0.08	3.43E-09	0.51	7.54E-04	0.21	4.78E-02
PsychENCODE Consortium	DR	ENSG00000087302	*C14orf166*	rs57496273	C	T	0.15	0.05	4.51E-05	-0.43	3.49E-97	-0.12	6.23E-05	0.66	1.09E-02
PsychENCODE Consortium	DR	ENSG00000015133	*CCDC88C*	rs1285847	C	T	0.48	0.04	1.81E-05	0.22	5.16E-24	0.16	7.93E-05	0.58	1.24E-02
PsychENCODE Consortium	DR	ENSG00000011114	*BTBD7*	rs11628518	T	C	0.16	-0.04	4.11E-05	-0.08	1.20E-09	0.56	6.74E-04	0.42	4.60E-02
PsychENCODE Consortium	DR	ENSG00000249839	*AC011330.5*	rs2957636	G	A	0.28	-0.03	6.85E-04	-0.53	6.47E-105	0.06	7.94E-04	0.36	4.94E-02
PsychENCODE Consortium	DR	ENSG00000157823	*AP3S2*	rs4932144	C	T	0.74	-0.08	2.26E-17	-0.16	4.57E-10	0.48	5.11E-07	0.06	4.18E-04

PsychENCODE Consortium	DR	ENSG00000260769	*RP11-491F9.5*	rs27920	A	T	0.59	-0.04	7.31E-06	0.17	3.31E-12	-0.25	1.63E-04	0.63	1.96E-02
PsychENCODE Consortium	DR	ENSG00000103479	*RBL2*	rs11859538	G	A	0.47	-0.03	6.81E-04	-0.26	4.59E-156	0.11	7.52E-04	0.25	4.78E-02
PsychENCODE Consortium	DR	ENSG00000050820	*BCAR1*	rs8062169	T	C	0.14	-0.08	8.40E-10	0.08	3.13E-10	-0.97	1.12E-05	0.06	3.43E-03
PsychENCODE Consortium	DR	ENSG00000267681	*CTD-3199J23.6*	rs11650272	G	A	0.36	0.05	1.04E-07	-0.32	6.67E-18	-0.14	5.99E-06	0.32	2.56E-03
PsychENCODE Consortium	DR	ENSG00000188554	*NBR1*	rs11650272	G	A	0.36	0.05	1.04E-07	-0.12	1.81E-16	-0.40	7.90E-06	0.39	2.98E-03
PsychENCODE Consortium	DR	ENSG00000225190	*PLEKHM1*	rs79724577	C	A	0.19	0.04	5.19E-05	-0.18	6.10E-17	-0.22	2.70E-04	0.07	2.68E-02
PsychENCODE Consortium	DR	ENSG00000264070	*DND1P1*	rs62063208	A	C	0.24	0.05	1.75E-06	-0.34	1.87E-19	-0.13	2.40E-05	0.51	5.76E-03
PsychENCODE Consortium	DR	ENSG00000185294	*SPPL2C*	rs17577369	G	A	0.24	0.05	5.07E-07	-0.25	9.69E-13	-0.20	4.00E-05	0.07	7.90E-03
PsychENCODE Consortium	DR	ENSG00000262500	*RP11-259G18.2*	rs199498	C	T	0.22	0.04	1.99E-05	-0.23	1.91E-10	-0.20	3.94E-04	0.45	3.31E-02
PsychENCODE Consortium	DR	ENSG00000261575	*RP11-259G18.1*	rs73984689	A	G	0.24	0.05	2.28E-06	-0.33	1.04E-13	-0.14	6.63E-05	0.08	1.10E-02
PsychENCODE Consortium	DR	ENSG00000238083	*LRRC37A2*	rs3874943	G	A	0.50	-0.05	7.28E-07	-0.72	1.15E-155	0.07	1.12E-06	0.19	8.40E-04
PsychENCODE Consortium	DR	ENSG00000232300	*FAM215B*	rs3874943	G	A	0.50	-0.05	7.28E-07	-0.40	1.67E-59	0.13	2.15E-06	0.10	1.11E-03
PsychENCODE Consortium	DR	ENSG00000073969	*NSF*	rs3874943	G	A	0.50	-0.05	7.28E-07	-0.13	2.36E-20	0.40	1.26E-05	0.18	3.76E-03
PsychENCODE Consortium	DR	ENSG00000263142	*LRRC37A17P*	rs4630591	T	C	0.21	0.05	2.24E-06	-0.16	2.28E-08	-0.30	3.05E-04	0.33	2.86E-02
PsychENCODE Consortium	DR	ENSG00000159202	*UBE2Z*	rs12939844	A	T	0.26	-0.05	2.21E-09	-0.17	6.63E-38	0.32	5.81E-08	0.12	8.16E-05
PsychENCODE Consortium	DR	ENSG00000005884	*ITGA3*	rs847696	A	G	0.09	-0.05	3.03E-05	-0.16	7.81E-10	0.31	5.57E-04	0.54	4.12E-02
PsychENCODE Consortium	DR	ENSG00000055483	*USP36*	rs55779573	C	T	0.52	-0.04	2.78E-08	0.19	8.12E-40	-0.23	3.05E-07	0.06	2.73E-04
PsychENCODE Consortium	DR	ENSG00000134769	*DTNA*	rs17557695	A	T	0.27	-0.03	2.06E-04	0.11	2.69E-20	-0.30	5.74E-04	0.24	4.21E-02
PsychENCODE Consortium	DR	ENSG00000141646	*SMAD4*	rs12964229	A	G	0.40	-0.04	1.26E-06	0.08	4.46E-08	-0.54	2.86E-04	0.15	2.75E-02
PsychENCODE Consortium	DR	ENSG00000166603	*MC4R*	rs11664167	A	T	0.34	0.05	5.00E-08	-0.11	1.15E-10	-0.41	3.15E-05	0.08	7.20E-03
PsychENCODE Consortium	DR	ENSG00000116014	*KISS1R*	rs2965285	G	A	0.07	0.07	2.61E-07	0.28	1.37E-08	0.23	1.37E-04	0.95	1.74E-02
PsychENCODE Consortium	DR	ENSG00000124164	*VAPB*	rs2268915	C	G	0.39	-0.03	4.79E-05	0.20	4.09E-41	-0.17	9.97E-05	0.55	1.44E-02
PsychENCODE Consortium	DR	ENSG00000183506	*PI4KAP2*	rs2072516	A	G	0.48	0.03	6.01E-04	0.42	2.05E-131	0.07	6.79E-04	0.25	4.60E-02
PsychENCODE Consortium	DR	ENSG00000100191	*SLC5A4*	rs57676160	G	A	0.04	0.06	2.65E-04	1.20	3.53E-84	0.05	3.37E-04	0.11	2.98E-02
PsychENCODE Consortium	DR	ENSG00000128335	*APOL2*	rs5756111	A	G	0.39	0.04	5.34E-04	-0.40	1.18E-78	-0.09	6.61E-04	0.94	4.60E-02
PsychENCODE Consortium	DR	ENSG00000100395	*L3MBTL2*	rs139452	C	G	0.36	-0.03	2.24E-04	-0.14	1.28E-22	0.21	5.55E-04	0.63	4.12E-02
whole blood eQTL-GTEX	AD	ENSG00000168918	*INPP5D*	rs6715767	A	G	0.43	0.01	1.59E-06	-0.17	5.63E-16	-0.07	3.65E-05	0.05	0.02069482
whole blood eQTL-GTEX	AD	ENSG00000172543	*CTSW*	rs645900	A	G	0.81	0.01	5.40E-06	0.35	5.43E-81	0.03	9.67E-06	0.16	0.01096928
whole blood eQTL-GTEX	AD	ENSG00000103811	*CTSH*	rs2289702	T	C	0.11	-0.01	5.18E-05	-0.65	5.71E-49	0.02	9.52E-05	0.42	0.03087935
whole blood eQTL-GTEX	AD	ENSG00000167394	*ZNF668*	rs4889530	A	T	0.40	0.01	1.09E-05	0.30	2.14E-21	0.03	6.58E-05	0.07	0.02488326
whole blood eQTL-GTEX	AD	ENSG00000103507	*BCKDK*	rs749671	A	G	0.38	0.01	9.84E-06	0.12	4.68E-14	0.08	1.37E-04	0.23	0.03884404
whole blood eQTL-GTEX	AD	ENSG00000234327	*AC012146.7*	rs56197642	G	C	0.11	0.02	1.26E-07	-0.38	3.04E-12	-0.05	2.53E-05	0.65	0.01721215
whole blood eQTL-GTEX	AD	ENSG00000104783	*KCNN4*	rs11879798	A	G	0.34	-0.01	5.49E-05	0.50	9.65E-62	-0.02	8.88E-05	0.36	0.03023158
whole blood eQTL-GTEX	AD	ENSG00000087589	*CASS4*	rs6014722	A	T	0.09	-0.02	2.16E-09	-0.25	1.79E-15	0.09	1.73E-06	0.11	0.00235236
whole blood eQTL-GTEX	DR	ENSG00000001460	*STPG1*	rs7536873	T	C	0.25	0.04	7.50E-05	-0.29	6.94E-12	-0.14	6.05E-04	0.18	2.79E-02
whole blood eQTL-GTEX	DR	ENSG00000090621	*PABPC4*	rs61779310	G	C	0.23	0.08	7.32E-13	-0.11	3.16E-11	-0.69	1.10E-06	0.66	3.38E-04
whole blood eQTL-GTEX	DR	ENSG00000227533	*SLC2A1-AS1*	rs71654267	A	G	0.16	0.05	2.62E-05	0.51	4.98E-23	0.09	1.09E-04	0.74	8.28E-03
whole blood eQTL-GTEX	DR	ENSG00000162437	*RAVER2*	rs567910619	T	C	0.01	-0.23	9.28E-05	2.50	2.78E-41	-0.09	1.74E-04	0.75	1.18E-02
whole blood eQTL-GTEX	DR	ENSG00000143363	*PRUNE*	rs140386498	T	A	0.02	-0.15	5.61E-05	0.49	2.88E-08	-0.31	1.11E-03	0.54	4.22E-02

whole blood eQTL-GTEX	DR	ENSG00000160781	*PAQR6*	rs6701440	G	A	0.46	0.03	1.83E-05	-0.71	0.00E+00	-0.05	2.06E-05	0.25	2.50E-03
whole blood eQTL-GTEX	DR	ENSG00000198952	*SMG5*	rs6701440	G	A	0.46	0.03	1.83E-05	-0.77	0.00E+00	-0.05	2.02E-05	0.21	2.50E-03
whole blood eQTL-GTEX	DR	ENSG00000163472	*TMEM79*	rs3762281	G	A	0.46	0.03	1.46E-04	-0.34	1.98E-58	-0.10	2.19E-04	0.06	1.34E-02
whole blood eQTL-GTEX	DR	ENSG00000198715	*C1orf85*	rs6701440	G	A	0.46	0.03	1.83E-05	-0.91	0.00E+00	-0.04	2.05E-05	0.17	2.50E-03
whole blood eQTL-GTEX	DR	ENSG00000135862	*LAMC1*	rs11588675	T	C	0.43	0.05	8.04E-10	-0.46	1.87E-39	-0.11	2.61E-08	0.37	1.35E-05
whole blood eQTL-GTEX	DR	ENSG00000117280	*RAB7L1*	rs708723	T	C	0.53	-0.03	7.33E-04	0.31	3.09E-41	-0.09	1.05E-03	0.17	4.08E-02
whole blood eQTL-GTEX	DR	ENSG00000162769	*FLVCR1*	rs2291771	C	T	0.19	-0.04	1.50E-05	0.64	1.71E-78	-0.07	2.47E-05	0.21	2.78E-03
whole blood eQTL-GTEX	DR	ENSG00000234936	*AC010883.5*	rs13399366	T	C	0.11	-0.12	1.18E-20	0.23	1.38E-08	-0.52	1.25E-06	0.12	3.41E-04
whole blood eQTL-GTEX	DR	ENSG00000170340	*B3GNT2*	rs11888817	C	T	0.65	0.04	1.34E-05	0.34	1.67E-74	0.11	2.29E-05	0.06	2.66E-03
whole blood eQTL-GTEX	DR	ENSG00000011523	*CEP68*	rs2080385	T	G	0.26	-0.04	6.42E-05	-0.42	1.60E-37	0.10	1.36E-04	0.11	9.86E-03
whole blood eQTL-GTEX	DR	ENSG00000253497	*IGKV1-13*	rs72848920	T	C	0.08	0.06	1.40E-04	1.17	6.07E-82	0.05	1.88E-04	0.49	1.22E-02
whole blood eQTL-GTEX	DR	ENSG00000073737	*DHRS9*	rs2302698	G	A	0.48	-0.03	6.76E-04	0.29	4.67E-54	-0.10	9.01E-04	0.20	3.70E-02
whole blood eQTL-GTEX	DR	ENSG00000178074	*C2orf69*	rs2163548	G	C	0.13	-0.05	4.17E-05	0.13	1.01E-08	-0.37	8.59E-04	0.69	3.64E-02
whole blood eQTL-GTEX	DR	ENSG00000064012	*CASP8*	rs3185378	C	G	0.61	0.04	2.69E-05	0.28	1.01E-60	0.12	4.74E-05	0.09	4.57E-03
whole blood eQTL-GTEX	DR	ENSG00000183625	*CCR3*	rs2201150	C	T	0.64	0.03	4.00E-04	0.42	3.33E-37	0.07	6.47E-04	0.40	2.93E-02
whole blood eQTL-GTEX	DR	ENSG00000163823	*CCR1*	rs2201150	C	T	0.64	0.03	4.00E-04	-0.39	7.67E-58	-0.08	5.46E-04	0.17	2.69E-02
whole blood eQTL-GTEX	DR	ENSG00000114650	*SCAP*	rs57346147	G	C	0.40	-0.03	5.67E-04	-0.13	7.07E-20	0.21	1.26E-03	0.08	4.65E-02
whole blood eQTL-GTEX	DR	ENSG00000178467	*P4HTM*	rs6442131	A	C	0.78	0.04	8.38E-05	0.11	1.67E-08	0.34	1.25E-03	0.16	4.65E-02
whole blood eQTL-GTEX	DR	ENSG00000198218	*QRICH1*	rs3087866	C	T	0.79	0.03	1.35E-04	-0.18	5.17E-16	-0.20	5.53E-04	0.38	2.69E-02
whole blood eQTL-GTEX	DR	ENSG00000242142	*SERBP1P3*	rs1864367	C	T	0.60	0.03	2.29E-04	0.46	1.06E-25	0.07	5.09E-04	0.60	2.61E-02
whole blood eQTL-GTEX	DR	ENSG00000163933	*RFT1*	rs1864367	C	T	0.60	0.03	2.29E-04	-0.13	1.20E-10	-0.24	1.38E-03	0.20	4.96E-02
whole blood eQTL-GTEX	DR	ENSG00000163946	*FAM208A*	rs61456176	A	G	0.04	0.07	2.47E-04	0.30	6.64E-24	0.22	5.71E-04	0.66	2.69E-02
whole blood eQTL-GTEX	DR	ENSG00000179021	*C3orf38*	rs4384978	C	G	0.84	0.05	4.34E-05	0.14	8.77E-11	0.36	5.42E-04	0.29	2.69E-02
whole blood eQTL-GTEX	DR	ENSG00000072858	*SIDT1*	rs35350834	A	G	0.33	-0.03	1.85E-04	-0.10	3.20E-13	0.34	8.80E-04	0.08	3.69E-02
whole blood eQTL-GTEX	DR	ENSG00000114544	*SLC41A3*	rs58582416	A	C	0.34	-0.03	6.26E-04	0.31	3.79E-35	-0.10	9.79E-04	0.41	3.95E-02
whole blood eQTL-GTEX	DR	ENSG00000163584	*RPL22L1*	rs3821747	G	A	0.23	-0.03	6.53E-04	0.33	1.58E-42	-0.10	9.42E-04	0.17	3.83E-02
whole blood eQTL-GTEX	DR	ENSG00000161204	*ABCF3*	rs843336	G	A	0.49	0.03	1.68E-04	0.12	1.83E-10	0.26	1.19E-03	0.64	4.48E-02
whole blood eQTL-GTEX	DR	ENSG00000259959	*RP11-121C2.2*	rs28460483	A	G	0.33	-0.03	1.12E-03	0.86	9.55E-81	-0.03	1.31E-03	0.60	4.76E-02
whole blood eQTL-GTEX	DR	ENSG00000170448	*NFXL1*	rs1371729	G	A	0.33	-0.03	8.82E-04	0.88	8.99E-122	-0.03	9.92E-04	0.39	3.97E-02
whole blood eQTL-GTEX	DR	ENSG00000170390	*DCLK2*	rs56150320	A	G	0.22	0.04	9.15E-05	0.71	4.21E-46	0.05	1.61E-04	0.43	1.12E-02
whole blood eQTL-GTEX	DR	ENSG00000164305	*CASP3*	rs72689254	T	C	0.12	-0.05	1.01E-04	-0.21	2.66E-17	0.22	4.11E-04	0.09	2.18E-02
whole blood eQTL-GTEX	DR	ENSG00000132842	*AP3B1*	rs252761	T	G	0.60	-0.03	7.29E-05	0.15	1.80E-19	-0.22	2.82E-04	0.21	1.64E-02
whole blood eQTL-GTEX	DR	ENSG00000152409	*JMY*	rs10052358	A	G	0.64	0.04	1.98E-05	-0.12	1.14E-14	-0.32	1.88E-04	0.31	1.22E-02
whole blood eQTL-GTEX	DR	ENSG00000145725	*PPIP5K2*	rs464720	T	C	0.28	0.03	1.05E-04	-0.28	1.14E-72	-0.12	1.49E-04	0.53	1.06E-02
whole blood eQTL-GTEX	DR	ENSG00000229390	*MICD*	rs2975031	G	A	0.69	-0.05	4.89E-07	0.45	5.67E-23	-0.10	7.40E-06	0.15	1.22E-03
whole blood eQTL-GTEX	DR	ENSG00000204625	*HCG9*	rs3116797	G	C	0.72	-0.05	8.66E-06	0.37	1.39E-15	-0.13	1.02E-04	0.46	7.87E-03
whole blood eQTL-GTEX	DR	ENSG00000238024	*DDX39BP2*	rs2245961	A	C	0.65	-0.04	9.65E-06	0.51	1.10E-28	-0.08	3.94E-05	0.10	3.97E-03
whole blood eQTL-GTEX	DR	ENSG00000204622	*HLA-J*	rs9260114	T	C	0.66	-0.04	2.05E-04	0.57	1.61E-32	-0.07	3.95E-04	0.21	2.11E-02
whole blood eQTL-GTEX	DR	ENSG00000137312	*FLOT1*	rs13197117	G	A	0.34	-0.04	3.34E-05	-0.09	8.38E-17	0.41	2.04E-04	0.43	1.28E-02

whole blood eQTL-GTEX	DR	ENSG00000137331	*IER3*	rs13197117	G	A	0.34	-0.04	3.34E-05	-0.32	9.48E-23	0.12	1.32E-04	0.23	9.71E-03
whole blood eQTL-GTEX	DR	ENSG00000204531	*POU5F1*	rs9380220	T	G	0.20	0.06	1.84E-07	0.37	1.27E-25	0.15	3.05E-06	0.07	6.85E-04
whole blood eQTL-GTEX	DR	ENSG00000204528	*PSORS1C3*	rs9380220	T	G	0.20	0.06	1.84E-07	0.63	3.88E-58	0.09	7.02E-07	0.07	2.57E-04
whole blood eQTL-GTEX	DR	ENSG00000271581	*XXbac-BPG248L*	rs9264802	T	C	0.17	-0.05	3.36E-05	0.95	4.31E-43	-0.05	7.16E-05	0.12	5.98E-03
whole blood eQTL-GTEX	DR	ENSG00000225851	*HLA-S*	rs2524096	T	G	0.45	-0.04	8.08E-06	0.91	4.14E-90	-0.05	1.32E-05	0.09	1.85E-03
whole blood eQTL-GTEX	DR	ENSG00000272221	*XXbac-BPG181B*	rs75098758	C	T	0.29	-0.04	6.40E-05	-0.86	2.40E-146	0.05	7.80E-05	0.46	6.34E-03
whole blood eQTL-GTEX	DR	ENSG00000204520	*MICA*	rs1131896	A	G	0.28	0.04	8.93E-06	0.46	2.65E-42	0.09	2.41E-05	0.23	2.75E-03
whole blood eQTL-GTEX	DR	ENSG00000224389	*C4B*	rs3132941	C	G	0.08	0.13	3.59E-11	1.17	1.85E-38	0.11	3.72E-09	0.07	2.51E-06
whole blood eQTL-GTEX	DR	ENSG00000229391	*HLA-DRB6*	rs28366333	C	G	0.24	0.06	9.62E-08	0.83	5.72E-90	0.07	2.53E-07	0.23	1.14E-04
whole blood eQTL-GTEX	DR	ENSG00000196735	*HLA-DQA1*	rs17843580	G	A	0.51	0.07	1.49E-15	-0.36	2.26E-67	-0.21	4.24E-13	1.00	9.53E-10
whole blood eQTL-GTEX	DR	ENSG00000223534	*HLA-DQB1-AS1*	rs9274639	C	T	0.73	0.08	3.14E-13	-0.51	2.15E-62	-0.15	2.42E-11	0.09	2.33E-08
whole blood eQTL-GTEX	DR	ENSG00000232629	*HLA-DQB2*	rs9274660	G	A	0.60	0.07	3.96E-14	0.97	0.00E+00	0.07	1.09E-13	0.80	3.69E-10
whole blood eQTL-GTEX	DR	ENSG00000224796	*RPL32P1*	rs1126509	A	T	0.30	0.05	3.52E-07	-0.23	2.91E-13	-0.19	2.96E-05	0.57	3.15E-03
whole blood eQTL-GTEX	DR	ENSG00000198937	*CCDC167*	rs6903910	A	G	0.55	-0.03	1.58E-04	0.17	3.37E-17	-0.19	5.65E-04	0.07	2.69E-02
whole blood eQTL-GTEX	DR	ENSG00000118520	*ARG1*	rs2781668	T	C	0.17	0.04	2.70E-04	-0.42	8.15E-71	-0.10	3.59E-04	0.31	1.98E-02
whole blood eQTL-GTEX	DR	ENSG00000112282	*MED23*	rs3843995	G	A	0.17	0.05	6.56E-05	-0.17	1.02E-19	-0.28	2.57E-04	0.89	1.54E-02
whole blood eQTL-GTEX	DR	ENSG00000026297	*RNASET2*	rs429083	C	T	0.51	0.03	1.05E-03	-0.42	4.42E-105	-0.06	1.20E-03	0.95	4.48E-02
whole blood eQTL-GTEX	DR	ENSG00000242435	*UPK3BP1*	rs62473744	G	A	0.81	-0.05	5.55E-05	-0.51	2.93E-15	0.09	3.30E-04	0.47	1.88E-02
whole blood eQTL-GTEX	DR	ENSG00000241852	*C8orf58*	rs755934	G	T	0.33	0.04	4.78E-05	0.22	1.10E-13	0.17	3.61E-04	0.34	1.98E-02
whole blood eQTL-GTEX	DR	ENSG00000158941	*CCAR2*	rs10099846	T	C	0.66	-0.04	7.15E-05	-0.12	3.26E-12	0.31	5.61E-04	0.59	2.69E-02
whole blood eQTL-GTEX	DR	ENSG00000147439	*BIN3*	rs1545837	C	T	0.37	0.04	1.94E-06	0.15	2.26E-17	0.27	3.32E-05	0.95	3.39E-03
whole blood eQTL-GTEX	DR	ENSG00000170791	*CHCHD7*	rs6474051	C	T	0.77	0.04	8.83E-05	0.20	1.37E-12	0.21	6.02E-04	0.72	2.79E-02
whole blood eQTL-GTEX	DR	ENSG00000164938	*TP53INP1*	rs896853	C	G	0.56	-0.03	3.38E-05	-0.11	8.17E-17	0.31	2.06E-04	0.78	1.28E-02
whole blood eQTL-GTEX	DR	ENSG00000147883	*CDKN2B*	rs662463	G	A	0.91	-0.06	1.37E-05	0.42	2.77E-25	-0.13	6.05E-05	0.68	5.37E-03
whole blood eQTL-GTEX	DR	ENSG00000165006	*UBAP1*	rs62556595	T	C	0.46	-0.04	1.11E-06	0.09	3.83E-13	-0.42	5.23E-05	0.34	4.97E-03
whole blood eQTL-GTEX	DR	ENSG00000213221	*DNLZ*	rs117527460	A	G	0.04	0.09	3.92E-05	1.55	2.77E-63	0.06	6.50E-05	0.05	5.69E-03
whole blood eQTL-GTEX	DR	ENSG00000148396	*SEC16A*	rs7020201	C	G	0.51	-0.03	6.04E-05	-0.35	3.54E-62	0.10	9.64E-05	0.51	7.65E-03
whole blood eQTL-GTEX	DR	ENSG00000187134	*AKR1C1*	rs67362530	A	G	0.13	-0.04	3.13E-04	0.95	1.00E-47	-0.05	4.69E-04	0.42	2.45E-02
whole blood eQTL-GTEX	DR	ENSG00000151632	*AKR1C2*	rs12764498	C	T	0.13	-0.04	2.57E-04	0.45	7.18E-20	-0.10	6.92E-04	0.35	3.09E-02
whole blood eQTL-GTEX	DR	ENSG00000181192	*DHTKD1*	rs9664990	A	G	0.20	0.05	3.14E-06	-0.12	1.86E-08	-0.43	3.32E-04	0.18	1.88E-02
whole blood eQTL-GTEX	DR	ENSG00000165626	*BEND7*	rs11258422	C	A	0.37	0.03	8.18E-05	0.26	2.83E-10	0.13	8.35E-04	0.05	3.60E-02
whole blood eQTL-GTEX	DR	ENSG00000107731	*UNC5B*	rs1124397	C	T	0.33	-0.04	2.85E-05	0.76	8.08E-51	-0.05	5.55E-05	0.66	5.17E-03
whole blood eQTL-GTEX	DR	ENSG00000075826	*SEC31B*	rs7900678	C	T	0.20	-0.03	7.61E-04	-0.55	2.64E-103	0.06	8.80E-04	0.73	3.69E-02
whole blood eQTL-GTEX	DR	ENSG00000166136	*NDUFB8*	rs4244338	G	A	0.19	-0.04	1.09E-04	-0.13	4.90E-14	0.29	5.77E-04	0.44	2.70E-02
whole blood eQTL-GTEX	DR	ENSG00000166135	*HIF1AN*	rs10883508	A	G	0.19	-0.04	2.34E-04	-0.33	1.06E-54	0.11	3.42E-04	0.81	1.91E-02
whole blood eQTL-GTEX	DR	ENSG00000107960	*OBFC1*	rs7100920	T	C	0.54	0.04	5.47E-07	0.18	1.47E-14	0.21	2.70E-05	0.05	2.99E-03
whole blood eQTL-GTEX	DR	ENSG00000177595	*PIDD*	rs7121646	A	T	0.54	-0.03	5.65E-04	0.18	2.47E-42	-0.17	8.30E-04	0.69	3.60E-02
whole blood eQTL-GTEX	DR	ENSG00000121691	*CAT*	rs34329347	G	A	0.35	-0.03	4.18E-04	0.54	1.06E-75	-0.06	5.29E-04	0.51	2.68E-02
whole blood eQTL-GTEX	DR	ENSG00000110025	*SNX15*	rs502333	A	G	0.71	-0.03	2.90E-04	0.26	2.37E-22	-0.12	6.85E-04	0.17	3.08E-02

whole blood eQTL-GTEX	DR	ENSG00000172977	*KAT5*	rs12281400	T	C	0.36	0.03	1.51E-04	0.13	9.66E-10	0.25	1.28E-03	0.12	4.66E-02
whole blood eQTL-GTEX	DR	ENSG00000172757	*CFL1*	rs7947929	A	G	0.66	-0.03	9.23E-05	0.07	3.25E-09	-0.49	1.10E-03	0.21	4.21E-02
whole blood eQTL-GTEX	DR	ENSG00000172803	*SNX32*	rs17854357	C	G	0.16	0.06	5.81E-08	0.74	9.17E-28	0.08	1.18E-06	0.44	3.41E-04
whole blood eQTL-GTEX	DR	ENSG00000254851	*RP11-109L13.1*	rs35186251	C	T	0.26	0.03	9.03E-04	1.01	2.52E-108	0.03	1.03E-03	0.26	4.05E-02
whole blood eQTL-GTEX	DR	ENSG00000149591	*TAGLN*	rs664082	G	C	0.74	-0.03	5.99E-04	0.81	2.30E-93	-0.04	7.12E-04	0.23	3.14E-02
whole blood eQTL-GTEX	DR	ENSG00000078237	*C12orf5*	rs3217860	G	A	0.26	0.06	9.68E-10	-0.20	2.80E-12	-0.30	4.19E-06	0.19	8.08E-04
whole blood eQTL-GTEX	DR	ENSG00000111642	*CHD4*	rs73264831	A	C	0.14	0.05	2.05E-06	-0.12	1.81E-08	-0.46	2.84E-04	0.30	1.64E-02
whole blood eQTL-GTEX	DR	ENSG00000166881	*TMEM194A*	rs62621200	T	C	0.02	0.09	4.18E-04	0.47	6.85E-20	0.20	9.97E-04	0.66	3.97E-02
whole blood eQTL-GTEX	DR	ENSG00000234608	*MAPKAPK5-AS1*	rs7965368	T	C	0.17	-0.04	9.61E-05	0.25	8.82E-14	-0.17	5.48E-04	0.34	2.69E-02
whole blood eQTL-GTEX	DR	ENSG00000112787	*FBRSL1*	rs77773608	A	C	0.30	0.04	7.87E-06	0.22	6.30E-23	0.18	4.70E-05	0.23	4.57E-03
whole blood eQTL-GTEX	DR	ENSG00000011114	*BTBD7*	rs12586312	C	T	0.19	-0.04	5.80E-05	-0.12	3.55E-09	0.33	8.90E-04	0.17	3.69E-02
whole blood eQTL-GTEX	DR	ENSG00000248508	*SRP14-AS1*	rs8041057	T	C	0.70	-0.05	1.33E-07	0.45	1.38E-34	-0.10	1.26E-06	0.16	3.41E-04
whole blood eQTL-GTEX	DR	ENSG00000173517	*PEAK1*	rs7119	T	C	0.41	0.04	1.64E-06	0.13	1.22E-09	0.30	1.68E-04	0.11	1.14E-02
whole blood eQTL-GTEX	DR	ENSG00000103811	*CTSH*	rs2289702	T	C	0.11	-0.06	1.78E-04	-0.65	5.71E-49	0.09	2.81E-04	0.88	1.64E-02
whole blood eQTL-GTEX	DR	ENSG00000166965	*RCCD1*	rs76119208	T	G	0.14	0.06	1.55E-06	0.28	4.33E-17	0.21	3.03E-05	0.29	3.15E-03
whole blood eQTL-GTEX	DR	ENSG00000213918	*DNASE1*	rs9989424	C	G	0.31	0.04	1.52E-05	-0.10	1.16E-08	-0.39	5.67E-04	0.52	2.69E-02
whole blood eQTL-GTEX	DR	ENSG00000103111	*MON1B*	rs7196424	G	C	0.56	-0.03	1.96E-04	0.14	2.48E-20	-0.22	5.52E-04	0.57	2.69E-02
whole blood eQTL-GTEX	DR	ENSG00000074755	*ZZEF1*	rs12452728	G	C	0.17	0.06	3.01E-08	0.13	1.64E-09	0.51	4.50E-05	0.19	4.47E-03
whole blood eQTL-GTEX	DR	ENSG00000198496	*NBR2*	rs2292595	C	G	0.36	0.04	2.03E-07	-0.56	1.15E-60	-0.08	7.24E-07	0.06	2.57E-04
whole blood eQTL-GTEX	DR	ENSG00000236383	*LINC00854*	rs799917	A	G	0.36	0.05	4.58E-07	-0.23	1.20E-10	-0.20	7.18E-05	0.26	5.98E-03
whole blood eQTL-GTEX	DR	ENSG00000175906	*ARL4D*	rs34053645	C	T	0.36	0.05	8.82E-08	-0.24	6.04E-09	-0.20	8.24E-05	0.58	6.62E-03
whole blood eQTL-GTEX	DR	ENSG00000264070	*DND1P1*	rs55974014	A	C	0.23	0.04	1.32E-05	1.32	1.64E-122	0.03	1.84E-05	0.11	2.38E-03
whole blood eQTL-GTEX	DR	ENSG00000263503	*RP11-707O23.5*	rs55974014	A	C	0.23	0.04	1.32E-05	1.24	3.51E-283	0.03	1.52E-05	0.40	2.09E-03
whole blood eQTL-GTEX	DR	ENSG00000214401	*KANSL1-AS1*	rs199456	T	C	0.22	0.04	5.03E-05	1.28	4.04E-291	0.03	5.59E-05	0.23	5.17E-03
whole blood eQTL-GTEX	DR	ENSG00000262500	*RP11-259G18.2*	rs17661015	C	T	0.24	0.05	5.94E-07	1.11	3.07E-131	0.04	1.00E-06	0.09	3.22E-04
whole blood eQTL-GTEX	DR	ENSG00000262539	*RP11-259G18.3*	rs2950015	G	A	0.24	0.05	3.12E-06	1.28	4.32E-302	0.04	3.72E-06	0.84	8.05E-04
whole blood eQTL-GTEX	DR	ENSG00000228696	*ARL17B*	rs62074125	C	A	0.23	-0.05	1.28E-06	0.75	4.23E-33	-0.06	7.11E-06	0.11	1.20E-03
whole blood eQTL-GTEX	DR	ENSG00000176681	*LRRC37A*	rs3874942	A	G	0.50	-0.05	7.26E-07	-0.69	8.40E-45	0.07	2.98E-06	0.07	6.85E-04
whole blood eQTL-GTEX	DR	ENSG00000002919	*SNX11*	rs3744347	A	G	0.77	0.03	8.12E-04	0.29	1.70E-50	0.10	1.08E-03	0.40	4.16E-02
whole blood eQTL-GTEX	DR	ENSG00000136436	*CALCOCO2*	rs603358	A	G	0.16	0.04	1.23E-04	-0.20	2.82E-09	-0.22	1.26E-03	0.24	4.65E-02
whole blood eQTL-GTEX	DR	ENSG00000243678	*NME1-NME2*	rs1062386	T	A	0.51	-0.03	1.79E-04	-0.23	1.02E-52	0.14	2.74E-04	0.59	1.62E-02
whole blood eQTL-GTEX	DR	ENSG00000229590	*MSX2P1*	rs2052083	T	C	0.28	-0.04	4.93E-05	-0.27	1.84E-13	0.14	3.79E-04	0.87	2.04E-02
whole blood eQTL-GTEX	DR	ENSG00000175221	*MED16*	rs55639032	T	C	0.25	-0.04	6.86E-05	-0.32	2.22E-38	0.12	1.42E-04	0.82	1.02E-02
whole blood eQTL-GTEX	DR	ENSG00000105607	*GCDH*	rs8012	G	A	0.52	-0.03	2.11E-04	0.30	4.18E-45	-0.10	3.39E-04	0.58	1.91E-02
whole blood eQTL-GTEX	DR	ENSG00000130479	*MAP1S*	rs35976034	T	G	0.58	-0.03	2.83E-04	-0.11	7.91E-12	0.27	1.34E-03	0.69	4.84E-02
whole blood eQTL-GTEX	DR	ENSG00000129933	*MAU2*	rs57962361	T	C	0.11	0.08	7.23E-11	-0.16	2.17E-15	-0.50	4.79E-07	0.34	1.90E-04
whole blood eQTL-GTEX	DR	ENSG00000256229	*ZNF486*	rs142492786	A	G	0.09	0.06	2.91E-05	-0.33	1.92E-14	-0.19	2.44E-04	0.16	1.48E-02
whole blood eQTL-GTEX	DR	ENSG00000104936	*DMPK*	rs62111759	T	A	0.47	-0.04	5.53E-08	0.19	1.29E-13	-0.24	1.18E-05	0.28	1.73E-03
whole blood eQTL-GTEX	DR	ENSG00000166704	*ZNF606*	rs10415951	C	T	0.38	-0.04	1.30E-05	0.12	4.00E-08	-0.31	6.39E-04	0.49	2.93E-02

whole blood eQTL-GTEX	DR	ENSG00000176593	*CTD-2368P22.1*	rs1014767	G	A	0.61	0.04	7.42E-06	-0.37	2.45E-31	-0.09	2.89E-05	0.45	3.14E-03
whole blood eQTL-GTEX	DR	ENSG00000101421	*CHMP4B*	rs2626562	A	G	0.52	0.03	1.10E-04	-0.22	1.88E-40	-0.13	2.04E-04	0.26	1.28E-02
whole blood eQTL-GTEX	DR	ENSG00000100311	*PDGFB*	rs59744664	G	A	0.39	-0.03	2.60E-04	-0.29	3.33E-14	0.10	1.00E-03	0.66	3.97E-02
whole blood eQTL-GTEX	DR	ENSG00000198355	*PIM3*	rs28645887	T	C	0.49	-0.03	4.80E-04	-0.26	6.00E-25	0.12	9.41E-04	0.80	3.83E-02

P , *P* -values from the corresponding analyses;

P HEIDI, *P* -value from the HEIDI test;

P FDR, *P* -value adjusted using the false discovery rate (FDR) method.

Table S6. MR estimates for the associations between DR, candidate mediators, and AD.

Exposure	Outcome	Method	N SNPs	OR	95%CI (lower)	95%CI (upper)	*P*- value
DR	Adiponectin	IVW	45	0.970	0.942	0.999	0.043
DR	ApolipoproteinB	IVW	91	0.948	0.893	1.008	0.088
DR	BMI	IVW	98	1.034	1.001	1.068	0.044
DR	Fasting blood glucose	IVW	44	1.089	1.052	1.127	<0.001
DR	Glutamate	IVW	44	1.017	1.001	1.033	0.038
DR	HbA1c	IVW	97	1.249	1.212	1.287	<0.001
DR	HDL	IVW	91	0.939	0.909	0.970	<0.001
DR	LDL	IVW	91	0.937	0.895	0.982	0.006
DR	MUFA	IVW	111	1.016	0.976	1.057	0.438
DR	Omega-3	IVW	111	0.988	0.955	1.022	0.480
DR	Omega-6	IVW	111	0.967	0.936	1.000	0.049
DR	Proinsulin	IVW	44	1.044	0.973	1.121	0.225
DR	PUFA	IVW	111	0.969	0.935	1.004	0.085
DR	SFA	IVW	111	1.000	0.963	1.039	0.987
DR	Triglycerides	IVW	91	1.041	0.994	1.090	0.092
Adiponectin	AD	IVW	11	0.993	0.955	1.033	0.729
ApolipoproteinB	AD	IVW	191	1.049	1.027	1.072	<0.001
BMI	AD	IVW	436	1.008	0.971	1.045	0.679
Fasting blood glucose	AD	IVW	14	1.041	0.998	1.087	0.063
Glutamate	AD	IVW	7	0.992	0.964	1.020	0.565
HbA1c	AD	IVW	326	1.004	0.990	1.017	0.597
HDL	AD	IVW	339	0.959	0.925	0.994	0.023
LDL	AD	IVW	176	1.049	1.008	1.093	0.019
MUFA	AD	IVW	62	0.996	0.959	1.034	0.833
Omega-3	AD	IVW	48	0.996	0.963	1.030	0.810
Omega-6	AD	IVW	60	1.035	0.969	1.106	0.307
Proinsulin	AD	IVW	8	0.976	0.948	1.005	0.107
PUFA	AD	IVW	65	1.027	0.970	1.087	0.360
SFA	AD	IVW	54	0.996	0.953	1.042	0.875
Triglycerides	AD	IVW	299	1.063	1.023	1.105	0.002

Table S7. Mediating role of each mediator in the causal associations between DR and AD.

Exposure	Outcome	Mediator	***β*** **mediation**	SE	Z	***P*-value**
DR	AD	Adiponectin	2.10E-04	6.14E-04	0.342	0.732
DR	AD	ApolipoproteinB	-2.53E-03	1.59E-03	-1.591	0.112
DR	AD	BMI	2.58E-04	6.36E-04	0.405	0.685
DR	AD	Fasting blood glucose	3.46E-03	1.99E-03	1.736	0.083
DR	AD	Glutamate	-1.70E-04	2.03E-04	-0.839	0.401
DR	AD	HbA1c	8.01E-04	1.52E-03	0.528	0.598
DR	AD	HDL	2.62E-03	1.34E-03	1.955	0.051
DR	AD	LDL	-3.12E-03	1.75E-03	-1.783	0.075
DR	AD	MUFA	-6.32E-05	3.10E-04	-0.204	0.838
DR	AD	Omega-3	4.95E-05	2.18E-04	0.228	0.820
DR	AD	Omega-6	-1.15E-03	1.27E-03	-0.906	0.365
DR	AD	Proinsulin	-1.05E-03	1.08E-03	-0.969	0.332
DR	AD	PUFA	-8.39E-04	1.04E-03	-0.808	0.419
DR	AD	SFA	-1.15E-06	6.88E-05	-0.017	0.987
DR	AD	Triglycerides	2.44E-03	1.64E-03	1.484	0.138

Table S8. Baseline Cox regression analysis.

Covariate	Level	Hazard ratio	95%CI (lower)	95%CI (upper)	*P*- value
DR	DR (baseline)	1.884	1.497	2.371	<0.001
Age	Age (per year)	1.233	1.223	1.244	<0.001
Sex	Sex: Female vs Male	0.924	0.849	1.006	0.068
BMI	BMI (kg/m^2)	0.983	0.974	0.993	0.001
BP	SBP (mmHg)	1.003	1.001	1.006	0.007
	DBP (mmHg)	0.991	0.987	0.996	<0.001
Smoking	Smoking: Yes vs No	1.036	0.958	1.120	0.377
Drinking	Drinking: Past vs Never	1.222	0.989	1.510	0.064
	Drinking: Current vs Never	0.677	0.580	0.789	<0.001
Race	Race: Black vs White	1.547	1.100	2.176	0.012
	Race: Other vs White	0.858	0.574	1.283	0.457
	Race: Asian vs White	0.570	0.397	0.816	0.002
HbA1c (mmol/mol)		1.010	1.007	1.013	<0.001
LDL (mmol/L)		1.006	0.964	1.050	0.776
HDL (mmol/L)		0.962	0.858	1.077	0.499

Table S9. Standardized mean differences of baseline covariates before and after inverse probability of treatment weighting in the diabetes-restricted cohort.

Covariate	Level	Type	Unweighted SMD	Weighted SMD	Absolute Weighted SMD
Age	Age (per year)	Continuous	0.084	0.045	0.045
Sex	Sex: Female vs Male	Categorical	-0.001	-0.002	0.002
BMI	BMI (kg/m^2)	Continuous	-0.005	0.015	0.015
BP	SBP (mmHg)	Continuous	0.142	0.007	0.007
	DBP (mmHg)	Continuous	-0.191	-0.008	0.008
Smoking	Smoking: Yes vs No	Categorical	-0.008	0.007	0.007
Drinking	Drinking: Past vs Never	Categorical	0.046	-0.003	0.003
	Drinking: Current vs Never	Categorical	-0.080	-0.001	0.001
Race	Race: Black vs White	Categorical	0.060	0.006	0.006
	Race: Other vs White	Categorical	0.037	0.005	0.005
	Race: Asian vs White	Categorical	0.045	-0.018	0.018
HbA1c (mmol/mol)		Continuous	0.565	0.056	0.056
LDL (mmol/L)		Continuous	-0.124	-0.018	0.018
HDL (mmol/L)		Continuous	0.056	-0.007	0.007
DM duration		Continuous	0.645	0.073	0.073

Table S10. Sensitivity analysis.

**Model**	**Covariate**	**Hazard ratio**	**95%CI (lower)**	**95%CI (upper)**	***P*- value**
Adjustment for UACR	DR (time-varying)	1.549	1.132	2.120	0.006
Landmark analysis	DR (landmark-defined)	2.178	1.445	3.281	<0.001
All-cause dementia	DR (baseline)	1.798	1.484	2.179	<0.001
Single imputation	DR (baseline)	1.825	1.498	2.223	<0.001
Adjustment for DM and DM duration	DR (time-varying)	1.218	0.871	1.704	0.248

Table S11. Landmark diagnostics.

Landmark time	Number at risk	Number of events	Incidence (%)	Log-rank *P*-value	HR (basic)	95% CI (basic)	*P*-value (basic)	HR (full)	95% CI (full)	*P*-value (full)	*P*-value (global PH test)
0.5	308052	2556	0.83	<0.001	2.51	2.01–3.14	<0.001	2.13	1.42–3.21	<0.001	0.13
1	307906	2553	0.83	<0.001	2.52	2.01–3.15	<0.001	2.15	1.42–3.23	<0.001	0.14
2	307294	2542	0.83	<0.001	2.50	2.00–3.13	<0.001	2.18	1.45–3.28	<0.001	0.17
3	306375	2524	0.82	<0.001	2.52	2.01–3.15	<0.001	2.18	1.44–3.28	<0.001	0.25
5	303992	2409	0.79	<0.001	2.42	1.91–3.05	<0.001	2.02	1.31–3.12	0.001	0.47

Table S12. Lag exclusion analysis.

Lag	Model	Hazard ratio	95%CI (lower)	95%CI (upper)	*P*- value
Lag 1 year	Baseline (basic)	2.301	1.890	2.800	<0.001
Lag 1 year	Baseline (full)	1.551	1.129	2.130	0.007
Lag 1 year	Time-varying (basic)	2.303	1.886	2.811	<0.001
Lag 1 year	Time-varying (full)	1.551	1.133	2.123	0.006
Lag 2 year	Baseline (basic)	2.293	1.882	2.794	<0.001
Lag 2 year	Baseline (full)	1.573	1.145	2.161	0.005
Lag 2 year	Time-varying (basic)	2.295	1.878	2.805	<0.001
Lag 2 year	Time-varying (full)	1.573	1.149	2.153	0.005
